# Thermal priming enhances yield after recurrent heat stress and involves SlHSFA2‐SlERFs in the regulation of transcriptional thermal memory in tomato

**DOI:** 10.1111/tpj.71162

**Published:** 2026-10-08

**Authors:** Pratima Debnath, Paula Korkuć, Krishna Kumar, Paula C. P. Bueno, Katja Jaeger, Christin Abel, Valentin Hennig, Nicole M. van Dam, Philip A. Wigge, Justyna J. Olas

**Affiliations:** ^1^ Leibniz Institute of Horticultural Sciences (IGZ) e.V. 14979 Großbeeren Germany; ^2^ Leibniz Institute for Zoo and Wildlife Research Alfred‐Kowalke‐Straße 17 10315 Berlin Germany; ^3^ Institute for Biodiversity, Ecology and Evolution (IBEE), Friedrich Schiller University Jena Dornburgerstraße 159 04473 Jena Germany

**Keywords:** transcriptional thermal memory, tomato, yield, priming, HEAT SHOCK FACTORs, ETHYLENE RESPONSE FACTORs

## Abstract

Heat stress (HS) severely limits plant growth and productivity, threatening agriculture and food security. Plants cope with recurrent HS through adaptive thermal memory, which allows them to retain a memory of prior moderate priming stress. However, while this process has been well studied in *Arabidopsis thaliana*, it remains unclear whether the same molecular mechanisms operate in horticultural crops such as tomato (*Solanum lycopersicum*). Here, we show that tomato plants display a longer thermal memory capacity than Arabidopsis. We found that thermal priming followed by recurrent HS enhanced yield of tomato plants under both controlled and field conditions. Consistent with previous reports, we show that HEAT SHOCK FACTOR A2 (SlHSFA2) is required for thermal priming, as *hsfa2* CRISPR mutants lost priming capacity. SlHSFA2 acts as positive regulator of a subset of type II transcriptional thermal memory genes, including *SlBAG6*. SlHSFA2 physically interacts with ETHYLENE RESPONSE FACTORs, which function as co‐activators of type II transcriptional thermal memory genes and further enhance the expression of those genes in response to heat. Together, these findings establish a regulatory module critical for heat adaptation in tomato.

## Significance Statement

Plants can retain a memory of previous heat exposure that improves their response to subsequent stress, but whether the underlying mechanisms are conserved in tomato and how thermal memory affects productivity under recurrent heat remains unclear. We show that tomato has a robust, long‐lasting thermal memory that enhances yield under controlled and field conditions, and identify an HSFA2–ERF regulatory module that regulates type II thermal memory genes, providing a mechanistic basis for crop resilience.

## INTRODUCTION

Plants are sensitive organisms that cannot escape the negative effects of high temperature. Therefore, they have evolved adaptive mechanisms, including thermal memory, that help them cope with sudden increases in temperature. By memorizing exposure to non‐lethal temperature stress (priming), *Arabidopsis thaliana* (Arabidopsis) plants can respond more effectively to subsequent high‐temperature stress (triggering) (Sedaghatmehr et al., 2016; Stief et al., 2014). This priming capacity lasts for 3–4 days, during which plants undergo transcriptional, metabolic, and physiological reprogramming to enhance their survival (Kappel et al., 2023; Larkindale et al., 2005; Ling et al., 2018; Olas et al., 2021; Sedaghatmehr et al., 2016; Serrano et al., 2019; Sharma et al., 2019). Once the memory phase, which is the interval between the initial and subsequent heat stress (HS), is exceeded, plants lose the ability to “remember” (Sedaghatmehr et al., 2016; Stief et al., 2014).

In Arabidopsis, transcriptional thermal memory is controlled by a conserved family of transcription factors (TFs) known as HEAT SHOCK FACTORs (HSFs), which activate the HS response (HSR) by regulating the expression of *HEAT SHOCK PROTEINs* (*HSPs*). This regulation occurs through binding to specific promoter sequences “heat shock elements” (HSE), thereby initiating thermotolerance responses (Scharf et al., 2012). Within the transcriptional thermal memory network only HSFA1s, HSFA2, HSFA3, and HSFA7b have been characterized in detail (Charng et al., 2007; Friedrich et al., 2021; John et al., 2024; Lämke et al., 2016; Oberkofler et al., 2025; Peng et al., 2025). HSFA2, HSFA3, and HSFA7b have been shown to be transcriptionally responsive to heat (John et al., 2024; Olas et al., 2021), whereas HSFA1s control thermal memory at the protein level (Peng et al., 2025; Sharma et al., 2019). HSFA1 proteins contain prion‐like domains and undergo phase separation in response to heat, forming transcriptionally active speckles that facilitate temperature‐dependent chromatin looping between HSFA1 and enhancers, enabling hyperinduction of type II transcriptional thermal memory genes (Peng et al., 2025). HSFA1a has been proposed as predominant regulator of thermal memory, activating downstream HSFs that acquire specific functions in this process (Nishizawa‐Yokoi et al., 2011; Yoshida et al., 2011). For instance, the importance of HSFA2 is required to maintain thermal memory capacity and hyperactivation of type II transcriptional thermal memory genes, its importance was highlighted by the loss of priming ability in *hsfa2* knockout mutants (Charng et al., 2007; Friedrich et al., 2021; Kappel et al., 2023; Lämke et al., 2016; Oberkofler et al., 2025). HSFA2 and HSFA3 can form complexes with each other and with additional factors to enhance the activation of heat‐responsive genes, including *HSP*s (Crawford et al., 2024; Friedrich et al., 2021; Kappel et al., 2023). HSFA7b regulates the expression of genes involved in ethylene signaling and biosynthesis, highlighting the importance of hormonal status and ethylene levels in thermal memory (John et al., 2024). Beyond these molecular factors, studies in Arabidopsis have revealed tissue‐specific regulation, splicing‐related memory, and involvement of sugars and hormones in thermal memory (John et al., 2024; Ling et al., 2018; Olas et al., 2021; Sharma et al., 2019; Yao et al., 2022).

While transcriptional thermal memory has been extensively characterized in Arabidopsis, it remains unclear whether similar mechanisms are conserved in horticultural crops such as tomato (*Solanum lycopersicum*) (Liu et al., 2025; Sun et al., 2018; Sun et al., 2019), one of the most widely cultivated and consumed crops worldwide. In contrast, the HSR has been studied in detail in tomato, revealing dependence on SlHSFA1a, SlHSFA2, and SlHSFB1 (Hahn et al., 2011), with SlHSFA1a acting as a master regulator. Whereas SlHSFA1a and SlHSFB1 are constitutively expressed, SlHSFA2 is heat‐inducible and becomes the most abundant HSF during recovery phase (Hahn et al., 2011; Scharf et al., 1998). Notably, SlHSFA2 interacts with SlHSFA1a to enhance the transcription of downstream targets, and the SlHSFA2‐SlHSFA1a complex exhibits strong activity than SlHSFA2 alone (Chan‐Schaminet et al., 2009).

Here, we show that tomato (*Solanum lycopersicum* cv. *Moneymaker*) has a strong capacity for thermal priming and retains thermal stress memory for longer than Arabidopsis. We identified type II transcriptional thermal memory genes, including SlHSFA2, and show that SlHSFA2 acts as an important regulator of transcriptional thermal memory as previously shown in Arabidopsis. RNA‐seq analysis on dissected shoot apices using *hsfa2* mutants after priming and triggering treatments revealed genes differentially regulated in the mutant compared with wild‐type (WT) plants, including transcriptional thermal memory genes type II such as *B‐CELL LYMPHOMA 2* (*Bcl‐2*)‐*ASSOCIATED ATHANOGENE 6* (*SlBAG6; Solyc01g095320*). We further showed that SlHSFA2 positively regulates the expression of *SlBAG6* and interacts with various ETHYLENE RESPONSE FACTORs (SlERFs) to enhance its activation. In contrast, SlERFs alone are not sufficient to activate the *SlBAG6* promoter. Finaly, we found that thermal priming after recurrent triggering stress increases fruit yield without altering the nutritional content, both under controlled and field conditions. This demonstrates that thermal priming represents a positive adaptive trait for plant productivity, offering a future perspective for breeding resilient tomato plants with enhanced yield.

## RESULTS

### Thermal priming improves tomato resilience and yield without affecting nutritional content

To investigate whether tomato (*Solanum lycopersicum* cv. Moneymaker) seedlings are primable and display thermal memory capacity, we grew tomato seedlings for 5 days on 0.5 × MS medium supplemented with 1% sucrose and subjected them first to a moderate stress (priming, 5 days after germination (DAG)) and, 3 days later, to a severe triggering stress (8 DAG), using a thermal memory assay previously established for Arabidopsis (Olas & Wigge, 2025). Triggered‐only (T, 8 DAG) plants displayed severe growth defects, similar to Arabidopsis, whereas previously primed plants (P + T) survived the triggering stress and continued to grow (60% green plants) (Figure S1A,B). Since the only difference between P + T and T‐only plants was that the P + T plants had previously received the priming treatment, this suggests that, as in Arabidopsis, priming also positively influences plant survival in tomato. Because thermal memory in Arabidopsis persists for only 3–4 days, we next investigated the duration of thermal memory in tomato. Five‐days‐old tomato seedlings were primed and subsequently exposed to triggering stress after a recovery phase of 5, 10, 20, or 25 days (Figure 1a; Figure S1A–E). P + T plants with a 3‐day memory phase showed 60% green plants and 40% plants with weak damage (Figure S1B). Extending the memory phase to 5 days reduced the proportion of fully green plants to 20% and increased the proportion of plants with weak damage (80%) (Figure 1b). P + T plants with a 10‐day memory phase showed a further reduction in survival rate, with 100% of the plants showing weak damage (Figure S1B,C), suggesting that the protective effect of priming gradually declines as the memory phase is extended. After a 20‐day memory phase, 40% tomato plants displayed severe damage and 60% were dead (Figure S1B,D). In contrast, all P + T plants with a 25‐day memory phase were dead (Figure S1B,E). These results indicate that the protective effect of priming progressively decreases with increasing memory phase duration and is eventually lost after 20 days.

**Figure 1:**
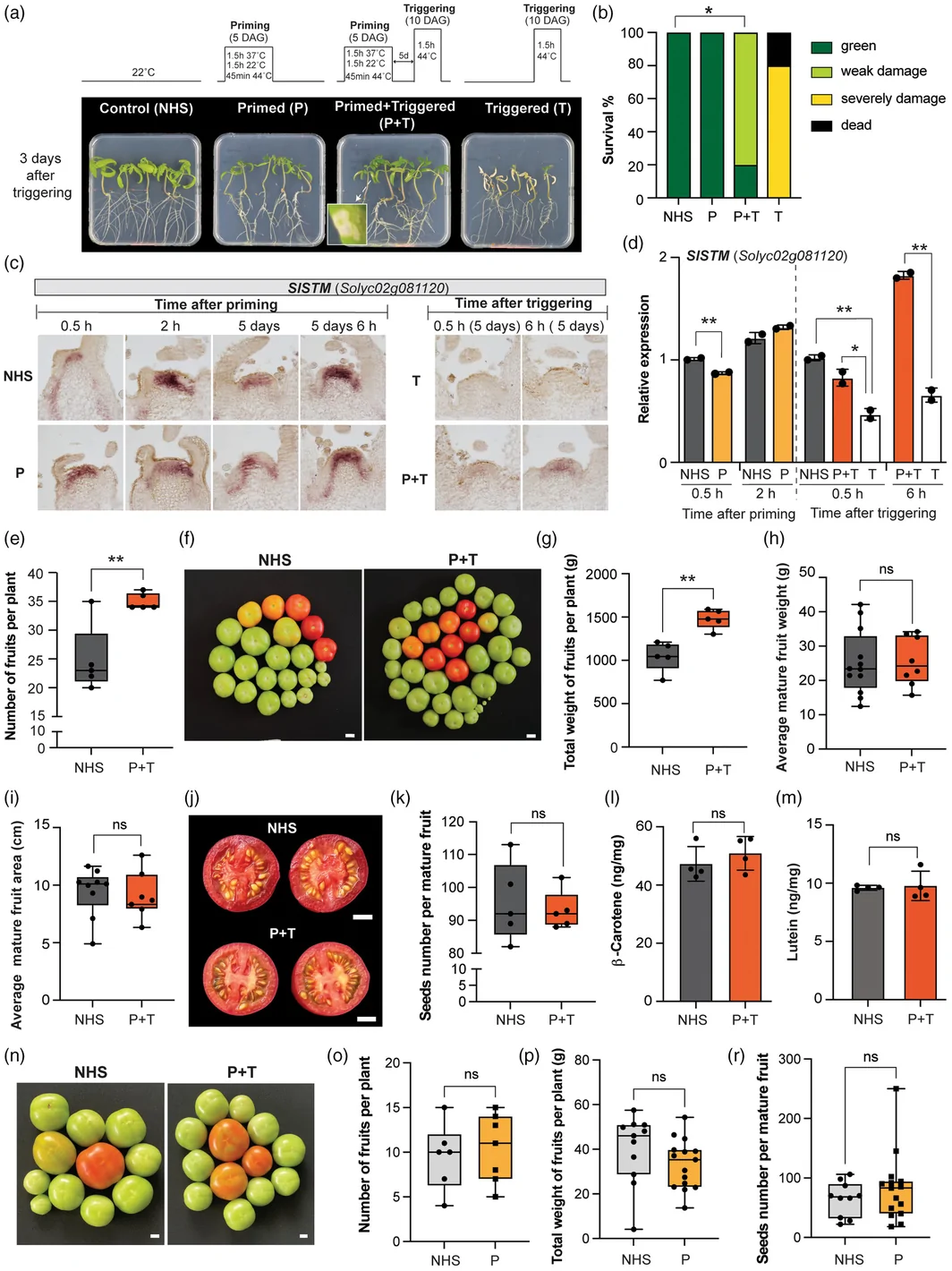
Thermally primed tomato plants subjected to recurrent triggering heat stress produce higher yield without affecting fruit quality in controlled growth conditions. (a) Phenotype of non‐treated (NHS), primed (P), primed + triggered (P + T), and triggered (T) tomato plants (*cv*. Moneymaker). Note that priming was performed at 5 days after germination (DAG), whereas triggering at 10 DAG. The memory phase was 5 days. Images were taken 3 days after triggering. (b) Phenotypic class distribution observed in the thermal memory assay shown in (a). (c) RNA *in situ* hybridization using *SHOOT MERISTEMLESS* (*STM*) as a probe on longitudinal sections through meristems from NHS, P, P + T, and T wild‐type plants. Samples were harvested at 0.5 h; 2 h, 5 days, and 5 days 6 h after priming, as well as at 0.5 h and 6 h after the triggering treatment. The 0.5 h time point following the triggering treatment corresponds to 120 h after the initial priming treatment. (d) Relative expression of *STM* analyzed by qRT‐PCR at 0.5 and 2 h after priming and at 0.5 and 6 h after triggering. (e) Average number of fruits per plant at 60 days after the first flower appeared in NHS and P + T plants grown in 22°C under long‐day (LD) conditions. (f) Representative images showing fruit number in NHS and P + T plants. (g) Average total fruit weight per plant in NHS and P + T plants. (h) Average mature fruit weight in NHS and P + T plants. (i) Average mature fruit area in NHS and P + T plants. (j) Representative images showing seed distribution in mature red fruits of NHS and P + T. (k) Average seed number per mature fruit in NHS and P + T plants. (l, m) β‐carotene and lutein levels measured in mature red fruits of NHS and P + T. (n) Representative images showing fruit number in NHS and P‐only plants. (o) Average number of fruits per plant at 60 days after the first flower appeared in NHS and P‐only plants grown in 22°C under LD conditions. (p) Average total fruit weight per plant in NHS and P‐only plants. (q) Average seed number per mature fruit in NHS and P‐only plants. Error bars indicate SD. Statistical significance was calculated using Student's *t*‐test *P < 0.05; **P < 0.01; ***P < 0.001. The experiment with NHS and P + T plants was performed twice, once experiment with NHS and P‐only plants once. Second independent experiment for NHS and P + T is described in Figure S2.

We then focused on a 5‐day memory phase for further analyses. Shoot fresh weight and shoot length were reduced in T‐only plants, whereas P + T plants showed no change in fresh weight compared with NHS controls and even significantly increased shoot length (Figure S1F,G). Thermal priming alone did not significantly affect shoot length in primed‐only plants but resulted in a reduction in shoot fresh weight. This suggests that the beneficial effects of thermal priming on shoot growth become apparent only after a subsequent triggering stress. Trypan blue staining 24 h after triggering stress revealed increased cell death in T‐only plants (Figure S1H), suggesting that triggering stress causes irreversible damage in the absence of priming. To examine meristem activity, we performed RNA *in situ* hybridization using the meristem maintenance gene *SHOOT MERISTEMLESS* (*SlSTM, Solyc02g081120*) as a probe (Clark et al., 1996) (Figure 1c). Priming alone did not alter *SlSTM* expression. In T‐only plants, however, *SlSTM* transcript was absent immediately after triggering stress and remained undetectable 6 h later, whereas in P + T plants it showed only transient downregulation and recovered after 6 h (Figure 1c). This indicates that priming protects meristem activity from severe HS, consistent with previous observations in *Arabidopsis* (Olas et al., 2021). qRT‐PCR analysis of the shoot apex confirmed that *SlSTM* and another meristem‐specific gene, *CLAVATA1* (*SlCLV1, Solyc04g081590*), recovered after an initial decline in P + T plants, whereas no recovery was observed in T‐only plants (Figure 1d; Figure S2A).

Because only P + T plants recovered meristem activity, we next assessed the effect of thermal priming on tomato growth and yield. Both P + T and NHS plants were transferred to soil three days after triggering (DAT) and placed in a controlled growth chamber (Figure 1e–m; Figure S2). No differences were observed in the timing of first flower appearance (Figure S2B). However, P + T plants produced significantly more fruits per plant than NHS plants, with an average increase of 10.2 ± 2.7 fruits (41% higher yield; *n* = 5) (Figure 1e,f). In addition, the total fruit weight of P + T plants was, on average, 432.1 ± 92.1 g per plant higher than that of NHS plants (Figure 1g), while no significant differences were observed in individual fruit weight, fruit area, and seed number among NHS and P + T plants (Figure 1h–k). A second independent experiment confirmed a yield increase of more than 30% in P + T plants (Figure S2C–F).

Since P + T plants produced more fruits, we next examined fruit quality. Levels of ß‐carotene and lutein were measured in red mature fruits, as both are health‐promoting compounds associated with reduced risk of chronic diseases (Collins et al., 2022) (Figure 1l,m). No significant differences were observed between P + T and NHS plants (Figure 1l,m), indicating that priming‐enhanced yield does not compromise nutritional composition.

To determine whether the increased yield of P + T plants was caused by the priming treatment alone or was specific to plants that experienced both priming and the subsequent triggering treatment (P + T), we grew primed‐only plants together with NHS plants under controlled growth chambers and analyzed fruit yield 60 days after the first flower had opened (Figure 1n–p). We found that primed‐only plants produced the same number of fruits as NHS plants (Figure 1n,o), indicating that the increased yield is specific to P + T plants and requires both the priming and triggering treatments. Height analysis of 1‐month‐old plants grown under controlled conditions showed that primed‐only plants were significantly shorter than NHS and P + T plants (Figure S2G,H).

Together, these findings demonstrate that tomato plants not only acquire heat priming capacity with a longer memory phase than Arabidopsis but also adaptively increase yield without affecting fruit quality.

### Thermal priming increases the yield in field grown plants

To determine whether the increased yield observed in P + T plants in controlled conditions (Figure 1) also occurs under naturally occurring, uncontrolled environmental conditions, we conducted a field experiment (Figure 2a). To provide environmental context, the average air temperature was recorded over the experiment (from July till September) using the weather station in the field as shown in Figure S3. NHS and P + T plants were transferred to pots 3 DAT (13 DAG) and moved to an open greenhouse at 10 DAT (20 DAG) for acclimatization (Figure 2a,b; Figure S4). Afterwards, they were transferred to the field at 17 DAT (27 DAG). By examining the shoot apices under a microscope and assessing the presence of flowers and flower primordia in NHS and P + T plants (*n* = 3), we confirmed that both groups were at the same developmental (reproductive) stage at this time (Figure 2b). Since primed‐only plants did not exhibit increased yield under controlled conditions, they were not included in the field experiment.

**Figure 2:**
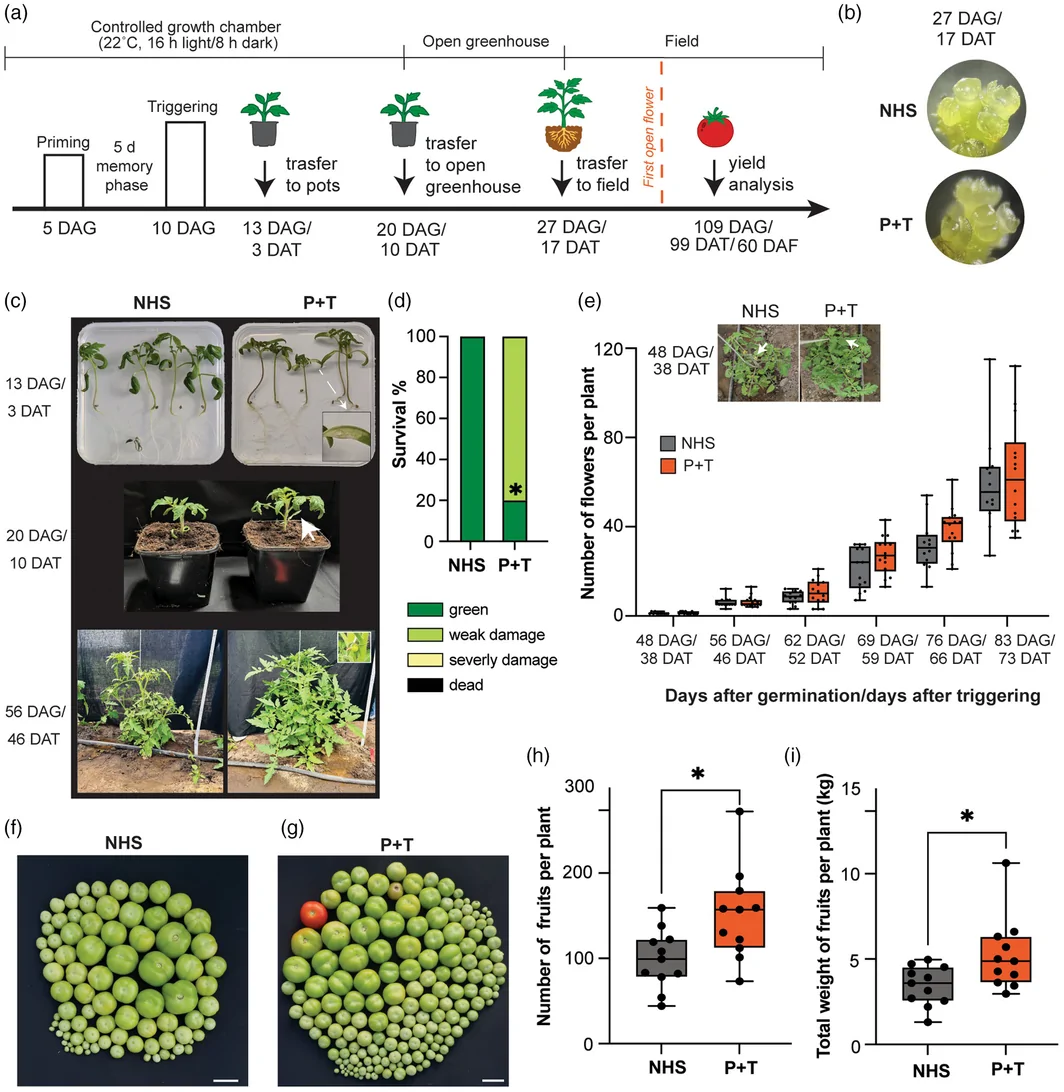
Thermal priming followed by triggering stress increases yield and enhance growth of tomato plants under non‐controlled growth conditions. (a) Experimental setup. Non‐heat‐stressed control (NHS) and primed + triggered (P + T) plants were transferred to the field at 27 days after germination (DAG), corresponding to 17 days after triggering (DAT). Yield analysis was performed 60 days after the appearance of the first flower (DAF). (b) Representative images of the shoot apical meristem of NHS and P + T plants on the day of field transfer (27 DAG/17 DAT). (c) Representative images of NHS and P + T plants during the course of the experiment. The magnified image shows signs of heat stress treatment in P + T plants. (d) Survival rate of NHS and P + T plants (13 DAG/3 DAT) after thermal memory assay shown in (c). Statistical difference was calculated using two‐sided Fisher’ exact text (*P* = 0.048). (e) Average number of flowers per plant in NHS and P + T plants, analyzed from 48 DAG/38 DAT to 83 DAG/73 DAT. Arrows show the flowers form by NHS and P + T. (f, g) Representative images of fruits produced by NHS (f) and P + T (g) plants. (h) Number of fruits produced by NHS and P + T plants. (i) Total fruit weight produced by NHS and P + T plants. Error bars indicate SD. Statistical significance was determined using Student's *t*‐test *P < 0.05. Sample sizes were *n* = 11 plants for both the NHS and P + T treatments. The field experiment was performed once under naturally occurring field conditions. Daily temperature records for the entire experimental period are provided in Figure S3.

The P + T plants exhibited visible HS symptoms, such as leaf bleaching at 3 DAT, which persisted after transfer to pots filled with soil (13 DAG) (Figure 2c,d). Interestingly, P + T plants developed taller shoots than NHS plants, with significant differences remaining even after transfer to the field and lasting up to 38 DAT (48 DAG) (Figure S5A). Moreover, at the time of transfer to the field, P + T plants displayed enhanced root growth (Figure S5B), as evidenced by significantly higher fresh and dry root weights (Figure S5C,D), indicating that priming promoted both below‐ and aboveground growth. We did not observe any differences in the timing of flower appearance between NHS and P + T plants grown in the field (see Figure 2e). However, yield analysis at 60 days after the first flowers appeared showed that P + T plants produced a significantly higher number of fruits per plant than NHS plants (Figure 2f–i), consistent with results from controlled conditions. On average, P + T plants produced 150.8 fruits per plant compared with 98.7 fruits in NHS plants, corresponding to an increase of 52.1 ± 19.3 fruits in P + T plants (Figure 2h). Moreover, the total fruit weight of P + T plants was also higher (5.232 kg) than that of NHS plants (3.437 kg), corresponding to an average increase of 1.795 ± 0.734 kg per plant (Figure 2i). In line with the experiment carried out under controlled conditions, no significant differences were observed in mature fruit diameter (Figure S5E) or seed number between NHS and P + T plants (Figure S5F,G).

Overall, we found that P + T plants produced a higher number of fruits in the field experiment, similar to what was observed under controlled conditions. However, it should be noted that the field experiment was conducted under naturally occurring conditions, in which temperature, light, humidity, etc. varied throughout the experiment and differed from conditions in the controlled growth chamber.

### Identification of the transcriptional thermal memory genes type II in tomato

To understand the molecular basis of transcriptional thermal memory and mechanisms that might contribute to the enhanced growth of P + T tomato plants, we performed RNA‐seq analysis on the hand‐dissected shoot apices from NHS and primed plants at 0.5 and 2 h after priming treatment, as well as from NHS, P + T, and T‐only plants at 0.5 h after triggering treatment (Figure 3a, Figure S6A,B; Table S1). Both P + T and T‐only plants were harvested at 10 DAG following an identical 1.5‐h triggering heat treatment, thus the only difference between the two groups was that the P + T plants had received a priming treatment 5 days earlier.

**Figure 3:**
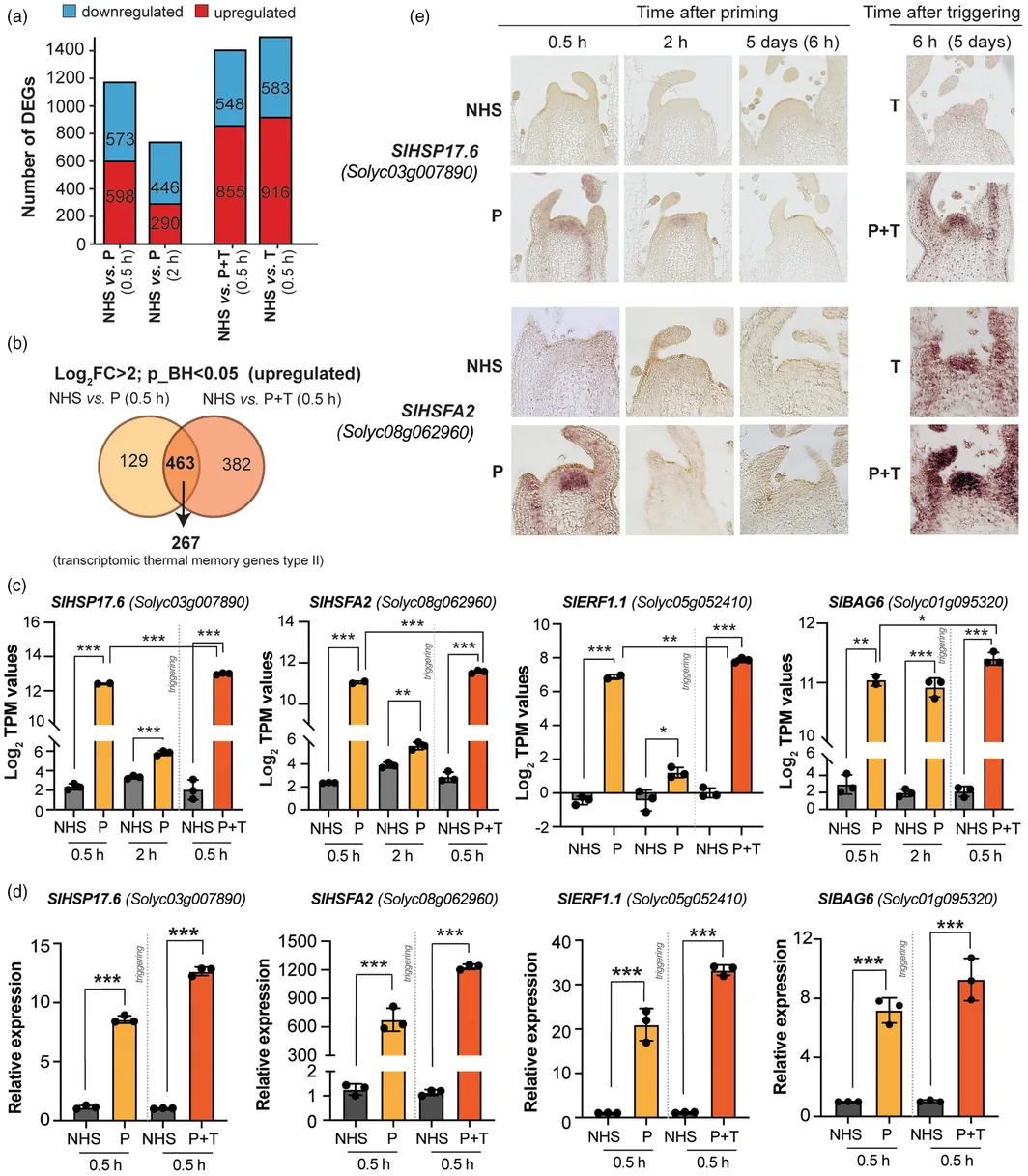
Expression of type II transcriptional thermal memory genes in the shoot apex of tomato. (a) Number of differentially expressed genes (DEGs) in the shoot apex of primed (P), primed + triggered (P + T), and triggered (T) tomato plants grown in 22°C under long‐day conditions (16 h light/ 8 h darkness) compared with non‐treated control (NHS) plants. Samples were analyzed at 0.5 and 2 h after priming and 0.5 h after triggering stress. (b) Venn diagram of upregulated DEGs (log_2_FC ≥ |2|; p_BH <0.05) in P versus NHS at 0.5 h after priming and in P + T versus NHS at 0.5 h after triggering. Note that 267 genes were hyperresponsive in P + T compared with P shoot apex and those defined as transcriptomic thermal memory genes type II. (c) Log_2_ TPM values of *HEAT SHOCK FACTOR A2 (SlHSFA2), HEAT SHOK PROTEIN* (*SlHSP17.6*) and *ETHYLENE RESPONSE FACTOR 1.1* (*SlERF1*) and *B‐CELL LYMPHOMA 2* (*Bcl‐2*)‐*ASSOCIATED ATHANOGENE* (*SlBAG6*) in the shoot apex of NHS and P plants at 0.5 and 2 h after priming, and in shoot apex of NHS and P + T plants at 0.5 h after triggering HS. Error bars indicate SD. (d) Relative expression of *SlHSFA2, SlHSP17.6, SlERF1*.*1*, and *SlBAG6* in the shoot apex of NHS and P plants at 0.5 h after priming, and in shoot apex of NHS and P + T plants at 0.5 h after triggering HS. Error bars indicate SD (*n* = 3). Statistical significance was calculated using Student's *t*‐test *P < 0.05; **P < 0.01; ***P < 0.001. (e) RNA *in situ* hybridization using *SlHSP17.6* and *SlHSFA2* as probes on longitudinal sections through meristems of NHS, P, P + T, and T plants collected at 0.5 h, 2 h and 5 days after priming, and 6 h after triggering. For NHS and P‐only plants, the 5‐day time point corresponds to the sampling time used for P + T and T plants at 6 h after triggering.

Based on our previous RNA‐seq analysis in Arabidopsis, which showed that primed plants are transcriptionally indistinguishable from NH plants at 72 h after priming (Olas et al., 2021), and because the triggering treatment was applied 5 days (120 h) after priming, we did not include primed‐only samples in the RNA‐seq analysis after triggering. To verify whether a similar response occurs in tomato, we analyzed the expression of the heat‐responsive genes *SlHSP17.6* and *SlHSFA2* during the memory phase at 0.5, 24, 72, and 120 h after priming in NHS and primed‐only plants. Both genes were strongly induced at 0.5 h after priming, but their expression returned to control levels by 24 h and remained comparable to NHS plants at 72 and 120 h after priming (Figure S6C,D). These results indicate that the transcriptional response to priming is transient and returns to basal levels before triggering treatment is applied, supporting the exclusion of a primed‐only plants from RNA‐seq.

Differentially expressed genes (DEGs), both up‐ and downregulated, were identified in the shoot apex after priming and triggering treatments in relation to NHS (Figure 2a; Table S1). In primed plants, 598 genes were upregulated (*p*_BH_ <0.05, log_2_FC ≥2) and 573 downregulated (*p*_BH_ <0.05, log_2_FC ≤−2) at 0.5 h after priming compared with NHS plants (Figure 3a). Similar to Arabidopsis, the number of DEGs in tomato declined rapidly after priming HS, with only 290 genes being upregulated and 446 downregulated 2 h after priming. In the shoot apex of P + T plants, comparison with the NHS controls identified 855 upregulated and 548 downregulated genes (Figure 3a). In T‐only plants, which were at the same developmental stage (10 DAG) and received identical triggering treatment, we identified 916 upregulated and 583 downregulated genes compared with NHS controls.

We next focused on identifying the transcriptomic thermal memory genes corresponding to type II transcriptional memory genes, as defined in previous studies (Balazadeh, 2022; Oberkofler et al., 2021; Pratx et al., 2024). In this context, type II transcriptional memory refers to genes that are induced (or repressed) during priming and exhibit enhanced transcriptional activation or repression (hyperresponsiveness) upon a subsequent triggering stress. We therefore compared the DEGs in primed‐only plants relative to NHS plants after priming as well as DEGs in P + T plants compared with NHS plants after triggering. We identified 4859 genes that were significantly upregulated 0.5 h after priming and again 0.5 h after triggering in P + T plants (pBH <0.05) (Figure S7A). To define a core set of transcriptomic thermal memory genes (type II), we applied an additional threshold of log_2_FC ≥1 (Figure S7B) and log_2_FC ≥2 (Figure 3b) together with pBH <0.05. This resulted in 862 (log_2_FC ≥1, pBH <0.05) and 463 (log_2_FC ≥2, pBH <0.05) genes that were upregulated after priming and triggering (Figure 3b; Figure S7B; Table S1). From these, 267 genes displayed higher expression in P + T plants than in primed plants and were classified as type II hyperresponsive transcriptomic thermal memory genes (Figure 3b). GO analysis revealed enrichment in categories such as “response to heat” (GO:0009408) and “response to temperature stimulus” (GO:0009628) (Table S2). Similarly, we found 59 transcriptomic downregulated thermal memory genes type II, including those associated with “Plant hormone signal transduction” (KEGG:04075) such as *ETHYLENE INSENSITIVE 3* (*Solyc01g006650*), *ERFC.2* (*Solyc04g014530*), *AUXIN‐INDUCED SAUR‐like PROTEIN* (*Solyc03g33590*) or *AUXIN*‐*REGULATED IAA22* (*Solyc06g008580*), as well as genes related to the cell wall (GO:0005618) (Table S2).

Among the most upregulated type II transcriptional thermal memory genes, we identified several *SlHSP*s, including *SlHSP17.6* (*Solyc03g007890*) (Figure 3c), *SlHSP70* (*Solyc11g066100*), and *SlHSP90* (*Solyc06g036290*) as well as *SlBAG5* (*Solyc06g072430*) and *SlBAG6* (*Solyc01g095320*) (Figure 3c) genes (Table S3). These genes are known to play important roles in thermotolerance both in tomato and Arabidopsis (Arif et al., 2021; He et al., 2025; Olas et al., 2021; Tichá et al., 2020; Yan et al., 2003), indicating that the meristem activates classical protective HS pathways similar to those described in Arabidopsis (Olas et al., 2021). In addition, several *SlHSFs* showed type II thermal memory patterns, with *SlHSFA2* (*Solyc08g062960*) and *SlHSFB2b* (*Solyc08g080540*) displaying the most pronounced transcriptional changes (Figure 3c; Figure S7C; Table S3). Furthermore, also genes involved in metabolic and hormonal pathways exhibit transcriptional thermal memory expression patterns. These include *SlERFs* such as *SlERF1.1* (*Solyc05g052410*) (Figure 3c), auxin‐responsive genes (*e.g. SAUR‐like auxin‐responsive family* (*Solyc04g082230*)), and *phosphoenolpyruvate carboxylase kinase* (*Solyc04g009900* and *Solyc09g090090*) (Table S3). This suggests that, as reported in Arabidopsis, hormonal regulation and carbon status may play similar roles in plant survival and transcriptional thermal memory in tomato.

We confirmed the hyperresponsiveness of *SlHSFA2*, *SlHSP17.6*, *SlERF1.1*, and *SlBAG6* by qRT‐PCR using *ACTIN* as the housekeeping gene (Figure 3d). To confirm the robustness of these results, we repeated the expression analysis using another housekeeping gene ‐ *SlCAC* (*Solyc08g006960*) (Figure S7D). The expression patterns obtained with *SlCAC* were consistent with those obtained using *ACTIN*, further confirming the hyperresponsiveness of these genes (Figure S7D). To gain cellular resolution of transcriptional thermal memory genes activation, we performed RNA *in situ* hybridization on longitudinal sections of WT meristems using *SlHSP17.6* and *SlHSFA2* as probes during thermal priming (Figure 3e). Consistent with the RNA‐seq results, both genes were strongly induced by priming and showed strong expression in the apex. Their expression was further hyperactivated in P + T plants following triggering stress, with stronger signals than in T‐only plants. Notably, *SlHSP17.6* and *SlHSFA2* transcripts were absent from the shoot apex under non‐stress conditions (Figure 3e), consistent with previous reports showing that *SlHSFA2* expression is barely detectable under control conditions in tomato (Fragkostefanakis et al., 2016).

### SlHSFA2 is required for transcriptional thermal memory in tomato

As *SlHSFA2* was among the most strongly induced type II transcriptional thermal memory genes in tomato (Figure S7C), and given that HSFs play an essential role in transcriptional thermal memory in Arabidopsis (Charng et al., 2007; Charng & Chen, 2023; Friedrich et al., 2021), we selected this factor for functional analysis.

We first analyzed the thermal response of previously characterized *hsfa2* CRISPR mutants lacking SlHSFA2 activity (Fragkostefanakis et al., 2016) (Figure 4a). Priming treatment had no visible effect on *hsfa2* mutants, which were indistinguishable from WT plants (Figure 4a,b). However, following triggering stress, P + T *hsfa2* plants displayed significantly reduced growth recovery compared with P + T WT plants (Figure 4a,b), indicating that SlHSFA2 is required for maintaining transcriptional thermal memory and priming capacity, similar as previously reported in Arabidopsis (Liu et al., 2013).

**Figure 4:**
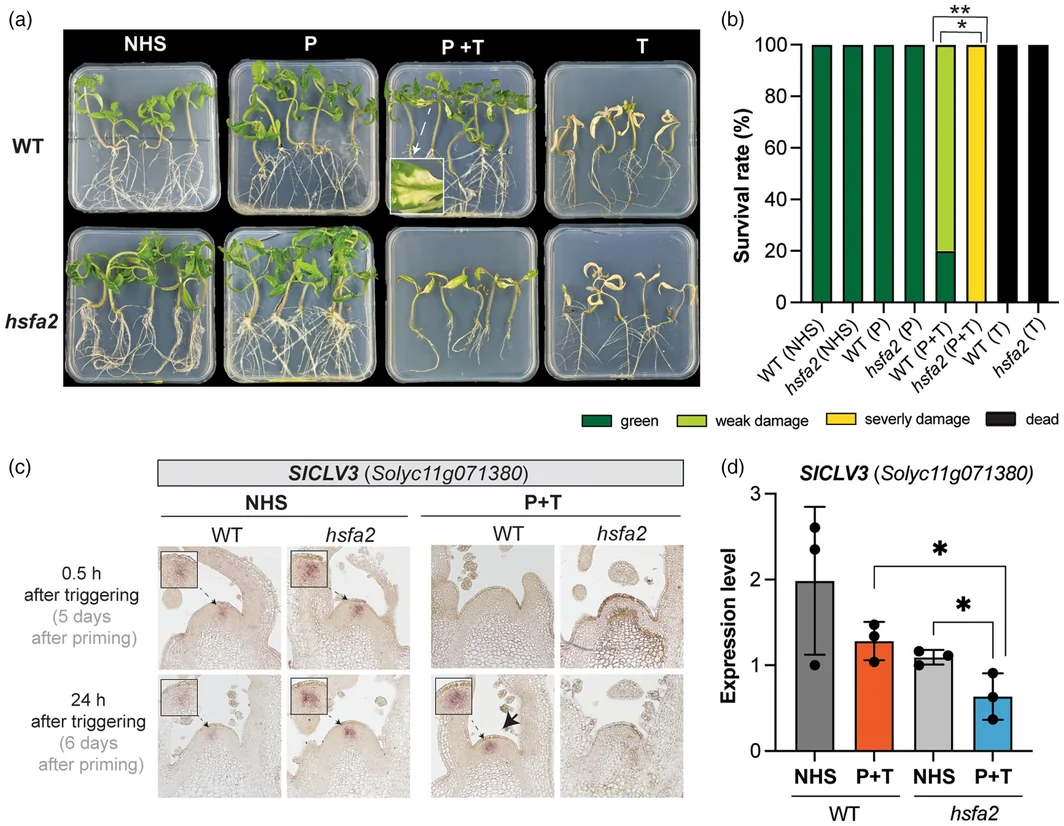
HEAT SHOCK FACTOR A2 (SlHSFA2) contributes to transcriptional thermal memory in tomato plants. (a) Thermopriming phenotype of non‐heat‐stressed (NHS), primed‐only (P), primed and triggered (P + T), and triggered‐only (T) wild‐type (WT) and *hsfa2* mutant plants. Images were taken 7 days after triggering. The magnified boxes show the bleached phenotype of P + T plants. (b) Phenotypic class distribution observed in the thermal memory assay shown in (a). Asterisks indicate significant differences compared with WT P + T plants (*P < 0.05, **P < 0.01, Fisher's exact test). (c) RNA *in situ* hybridization using *CLAVATA 3* (*SlCLV3*) as a probe on longitudinal sections through the meristems of NHS and P + T WT and *hsfa2* mutant tomato plants at 0.5 and 24 h after triggering. (d) Relative expression of *SlCLV3* analyzed at the shoot apex of NHS and P + T WT and *hsfa2* mutant plants at 0.5 h after triggering. Error bars indicate SD (*n* = 3). Statistical significance was calculated using Student's *t*‐test *P < 0.05.

To test this further, we examined the expression of stem cell regulator *SlCLV3* (*Solyc11g071380*) in the meristem of WT and *hsfa2* mutant plants by RNA *in situ* hybridization (Figure 4c). In NHS WT and *hsfa2* mutants, *SlCLV3* expression was comparable. In contrast, in P + T WT plants, *SlCLV3* expression declined 0.5 h after triggering HS but recovered by 24 h. In contrast, no recovery of *SlCLV3* was observed in P + T *hsfa2* mutants (Figure 4c), demonstrating that meristem activity was fully inhibited by triggering stress and that *hsfa2* mutants were lethal, consistent with the phenotypic observations (Figure 4a,b). qRT‐PCR analysis confirmed a stronger reduction of *SlCLV3* and *WUSCHEL* (*SlWUS*) transcripts in P + T *hsfa2* mutants compared with P + T WT plants (Figure 4d; Figure S8), further supporting the essential role of SlHSFA2 in transcriptional thermal memory.

To assess if SlHSFA2 is involved in the regulation of the expression of type II transcriptomic thermal memory genes, we examined how many of the identified transcriptional thermal memory genes in the WT tomato shoot apex contain a HSE motif (‐GAANNTTC‐) in their promoter regions (Tables S1, S5, S7), potentially making them targets of HSF‐mediated regulation, including SlHSFA2. To this end, we searched for the HSE motif within 1 kb upstream of the annotated transcription start site (TSS) in 267 upregulated and 59 downregulated transcriptional thermal memory genes. A significant enrichment of HSE motifs was observed among upregulated transcriptional thermal memory genes (105 genes with HSE; *P* = 0.0016, Fisher's exact test) (Table S5), suggesting that these genes may be directly bound by HSFs, including SlHSFA2. In contrast, no enrichment was observed among downregulated genes (*P =* 0.48, Fisher's exact test) (Table S6). These results support a model in which HSFs, including SlHSFA2, might directly bind to and transcriptionally activate a subset of type II transcriptional thermal memory genes.

To determine the role of SlHSFA2 in regulating type II transcriptional thermal memory genes, we performed RNA‐seq on shoot apices from NHS and P + T WT and *hsfa2* plants collected 0.5 h after triggering stress (Figure 5a; Figure S9). Principal component analysis (PCA) revealed that one biological replicate from the *hsfa2* P + T group clustered more closely with the WT P + T samples than with the other *hsfa2* P + T replicate (Figure S9A). Both biological replicates were kept for further analysis. To increase the robustness of the analysis and identify thermal memory genes more confidently dependent on SlHSFA2, we applied a more stringent differential expression threshold. Using the standard cutoff (pBH <0.05; log_2_FC ≥ |1|), we identified 3048 significantly DEGs in WT P + T versus WT NHS and 2825 DEGs in P + T *hsfa2* versus NHS *hsfa2* plants (Figure 5a, Figure S9B; Table S7). Applying a more stringent cutoff (pBH < 0.05; log_2_FC ≥ |2|), we identified 916 DEGs in P + T WT versus NHS WT (234 upregulated and 682 downregulated genes) and 905 significant DEGs in P + T *hsfa2* versus NHS *hsfa2* (299 upregulated and 606 downregulated) at 0.5 h after triggering (Figure S9C).

**Figure 5:**
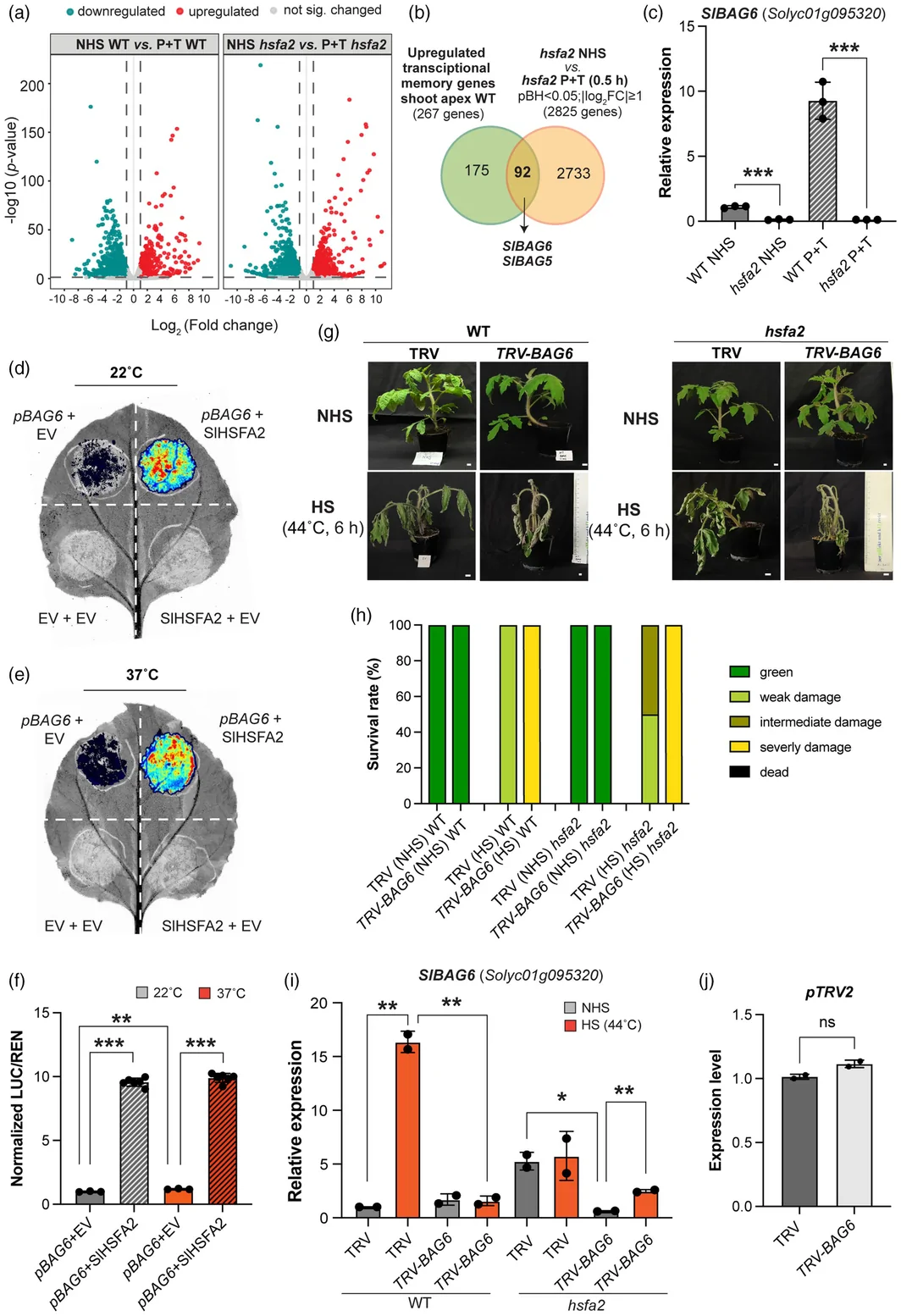
SlHSFA2 positively regulates the expression of a set of type II transcriptional thermal memory genes including *SlBAG6*. (a) Volcano plots of differentially expressed genes (DEGs) in non‐heat‐stressed (NHS) wild‐type (WT) versus P + T WT and NHS *hsfa2* versus P + T *hsfa2* showing the |log_2_ fold change| (*x*‐axis) and ‐log10 *P*‐value (*y*‐axis) of each gene. (b) Venn diagram of 267 transcriptional upregulated thermal memory genes identified in the shoot apex of WT plants and DEGs in WT *hsfa2* versus P + T *hsfa2* at 0.5 h after triggering. (c) Relative expression of *B‐CELL LYMPHOMA 2* (*Bcl‐2*)‐*ASSOCIATED ATHANOGENE* 6 (*SlBAG6*) analyzed at the shoot apex of NHS and P + T WT and *hsfa2* mutant plants at 0.5 h after triggering. Error bars indicate SD (*n* = 3). (d, e) Luciferase reporter assay showing activation of *SlBAG6* promoter by SlHSFA2 in *N. benthamiana* leave in (d) 22°C and (e) 37°C. (f) The quantifications of the luciferase activity presented in (d, e) as LUC/REN. (g) Phenotype of *SlBAG6*‐silenced WT and *hsfa2* plants exposed to 22°C (NHS) and heat stress (44°C) for 6 h. Scale bar 1 cm. (h) Survival rate of plants shown in panel (g). *n* = 2. (i) Expression level of *SlBAG6* in *SlBAG6*‐silenced WT and *hsfa2* plants analyzed by qRT‐PCR using leaf tissues. Error bars indicate SD (*n* = 2). Statistical significance was calculated using Student's *t*‐test *P < 0.05; **P < 0.01; ***P < 0.001. (j) Expression level of *pTRV2* in TRV‐EV and pTRV2‐*BAG6* (WT) plants under non‐stressed conditions showed no significant (ns) change. Error bars indicate SD (*n* = 2).

Since we were particularly interested in whether SlHSFA2 regulates type II transcriptional thermal memory genes, we compared the 267 upregulated transcriptional thermal memory genes identified in the shoot apices of WT tomato plants (of which 105 genes contained HSE motifs, see Figure 3) with the 2825 DEGs identified in P + T *hsfa2* mutants versus NHS *hsfa2* plants (pBH <0.05; log_2_FC ≥|1|) (Figure 5b). We found that 92 upregulated type II transcriptional thermal memory genes were differentially expressed in the *hsfa2* mutant, including *SlBAG5*, *SlBAG6*, and *SlHSP17.6* (Figure 5b). We examined whether all 92 commonly identified genes showed hyperresponsiveness in WT shoot apices in a second independent RNA‐seq analysis. We found that 9 of these 92 genes did not display this pattern and were therefore excluded from further analysis. To visualize differences in the expression patterns of the remaining 83 genes between P + T WT and *hsfa2* mutant plants, we generated a heatmap comparing their transcriptional profiles (Figure S10). This analysis revealed that heat‐induced expression was substantially reduced for many type II transcriptional thermal memory genes in P + T *hsfa2* mutants compared with P + T WT plants (Figure S10), indicating that SlHSFA2 contributes to the heat‐induced transcriptional response of a substantial subset of these genes. Among these potentially SlHSFA2‐dependent genes were *SlBAG5*, *SlBAG6, SlHSP17.6*, and *SlERF1.1* (Figure S10).

qRT‐PCR analysis on shoot apices of NHS and P + T WT and *hsfa2* mutants confirmed the reduced expression of *SlBAG6*, *SlBAG5, SlHSP1,7.6*, and *SlERF1.1* in P + T *hsfa2* mutants compared with P + T WT plants (Figure 5c, Figure S11), indicating that the transcriptional activation of these genes at the SAM in response to triggering HS might involve SlHSFA2. This was particularly evident for *SlBAG6* and *SlBAG5* (Figure 5c; Figure S11), where heat‐induced transcription was abolished in the absence of functional SlHSFA2, indicating that SlHSFA2 is involved in the regulation of heat‐induced transcription of these two genes.

Next, we tested whether SlHSFA2 can activate the promoter of selected transcriptional thermal memory genes, including *SlBAG6* by performing a luciferase reporter assay in *N. benthamiana* leaves at 22°C and 37°C (Figure 5d,e). We found that SlHSFA2 activates the *SlBAG6* promoter in transient expression assays (Figure 5d–f), indicating that SlHSFA2 functions as a positive regulator of *SlBAG6* expression. As a control, we also tested the *SlERF1.1* promoter, which expression was significantly reduced in P + T *hsfa2* mutants compared with P + T WT plants, but which does not contain any identifiable HSEs (Figure S12). In contrast, SlHSFA2 did not activate the *SlERF1.1* promoter in transient expression assay, indicating that SlHSFA2 might regulates only a subset of transcriptional thermal memory genes type II and that additional regulatory factors contribute to the regulation of transcriptional thermal memory.

To support our model that SlHSFA2 is involved in the regulation of the transcription of *SlBAG6* in plants, we transiently silenced *SlBAG6* using a virus‐induced silencing (VIGS) system in the both WT and *hsfa2* mutant backgrounds (Figure 5g). Because the TRV‐based VIGS system cannot be efficiently implemented in the young seedlings used for the thermal memory assays in this study, the functional analysis of VIGS‐silenced plants was performed using an acute HS treatment (44°C) for 6 h. Silencing *SlBAG6* in WT plants resulted in severe damage following HS compared with the empty vector (EV) controls, while no phenotype was observed under non‐stressed conditions (Figure 5g,h). Furthermore, we observed that *SlBAG6*‐silenced plants in the *hsfa2* background showed a phenotype similar to that of *SlBAG6*‐silenced plants in the WT background, whereas EV‐*hsfa2* plants exhibited a less severe phenotype (Figure 5g,h). This indicates that the heat‐sensitive phenotype caused by loss of SlHSFA2 is largely mediated through *SlBAG6*, supporting a genetic dependency. We next measured *SlBAG6* expression in *SlBAG6*‐silenced plants in both backgrounds by qRT‐PCR in leaf tissue (Figure 5i). The heat‐induced induction of *SlBAG6* transcript observed in pTRV2‐EV plants was only reduced in *pTRV2*‐*BAG6* silenced plants in both the WT and *hsfa2* backgrounds. qRT‐PCR revealed comparable *pTRV2* expression (Figure 5j), confirming silencing specificity.

Together with the qRT‐PCR analyses performed at the shoot apices of primed + triggered *hsfa2* mutants, these results point a tissue‐specific regulation of *SlBAG6* by SlHSFA2. At the SAM, SlHSFA2 appears to act as the predominant regulator of *SlBAG6* expression, whereas in leaves *SlBAG6* expression is likely to be controlled by additional transcriptional regulators.

### HSFA2 interacts with ERFs to enhance the expression of *SlBAG6*

Since many ethylene‐related genes, including several *SlERFs*, were differentially expressed in P + T WT plants compared with NHS WT plants after triggering stress (Table S1), with some (e.g. *SlERF1.1*) displaying type II transcriptional thermal memory expression patterns, we hypothesized that SlERFs might participate in transcriptional thermal memory. Therefore, we examined their relationship with SlHSFA2, which we identified to be involved in regulation of type II transcriptional thermal memory in tomato.

First, we analyzed expression changes of *SlERFs* and other ethylene‐related genes in NHS WT, P + T WT, NHS *hsfa2*, and P + T *hsfa2* plants at 0.5 h after triggering stress by generating a heatmap showing log_2_FC values relative to NHS plants (Figure S13). We confirmed the expression changes in P + T WT plants compared with NHS WT plants. However, the expression of these genes was largely unaffected in the absence of SlHSFA2 (NHS *hsfa2* vs. P + T *hsfa2*; Figure S13). Together with the finding that SlHSFA2 did not activate the *SlERF1.1* promoter (Figure S12), these results led us to hypothesize that SlHSFA2 is not directly involved in regulating *SlERFs* transcription during thermal priming. Instead, SlHSFA2 may interact with SlERFs proteins to regulate transcriptional thermal memory.

To test this, we first performed subcellular localization studies using *35S::HSFA2‐mTurquoise* and several SlERF proteins fused to *mVenus*. These included transcriptional thermal memory gene type II – *SlERF1.1*, genes significantly differentially expressed only in NHS WT versus P + T WT, such as *SlERF1a* (*Solyc03g093540*) and *SlERF.C.6* (*Solyc03g093560*); and genes significantly differentially expressed in both NHS WT versus P + T WT and NHS *hsfa2* versus P + T *hsfa2*, including *SlERF4* (*Solyc12g009240*), *SlERF1*.2 (*Solyc08g066660*), *AP2‐like ethylene‐responsive transcription factor* (*Solyc06g068570, SlAP2‐like factor*), *MULTIPROTEIN BRIDGING FACTOR 1‐ Ethylene‐Responsive 24 (Solyc01g104740, SlMBF1‐ER24*), and *SlERF1b* (*Solyc01g090340*) (Figure S14A; Table S7). By transiently expressing these constructs in *N. benthamiana* leaves at 37°C, we found that SlHSFA2 co‐localized in the nucleus with all tested proteins (Figure S14A).

To test whether SlHSFA2 interacts with SlERFs *in vitro*, we performed a yeast two‐hybrid assay examining the interaction between SlHSFA2 and selected SlERFs including SlERF1.1, SlERF1a, SlERF1b, SlERF4, and SlERF.C.6 (Figure 6a). Yeast co‐expressing BD‐SlHSFA2 and AD‐SlERFs grew on selective SD–Leu–Trp–His/‐–Ade medium, indicating interaction (Figure 6a). No growth was observed for the AD‐SlERFs with empty BD vector (Figure S14B). To examine whether these interactions also occur *in planta*, we carried out a bimolecular fluorescence complementation assay in *N. benthamiana* leaves at 37°C. For this, we generated constructs in which SlHSFA2 and individual SlERFs, SlAP2‐like factor, and SlMBF1‐ER24 were fused to the N‐terminal and C‐terminal fragments of YFP, respectively, under the control of the 35S promoter (Figure 6b; Figure S14C). We detected an YFP fluorescence signal for SlHSFA2 together with all tested SlERFs (Figure 6b; Figure S14C), supporting our model that SlHSFA2 physically interacts with multiple SlERF proteins *in planta*.

**Figure 6:**
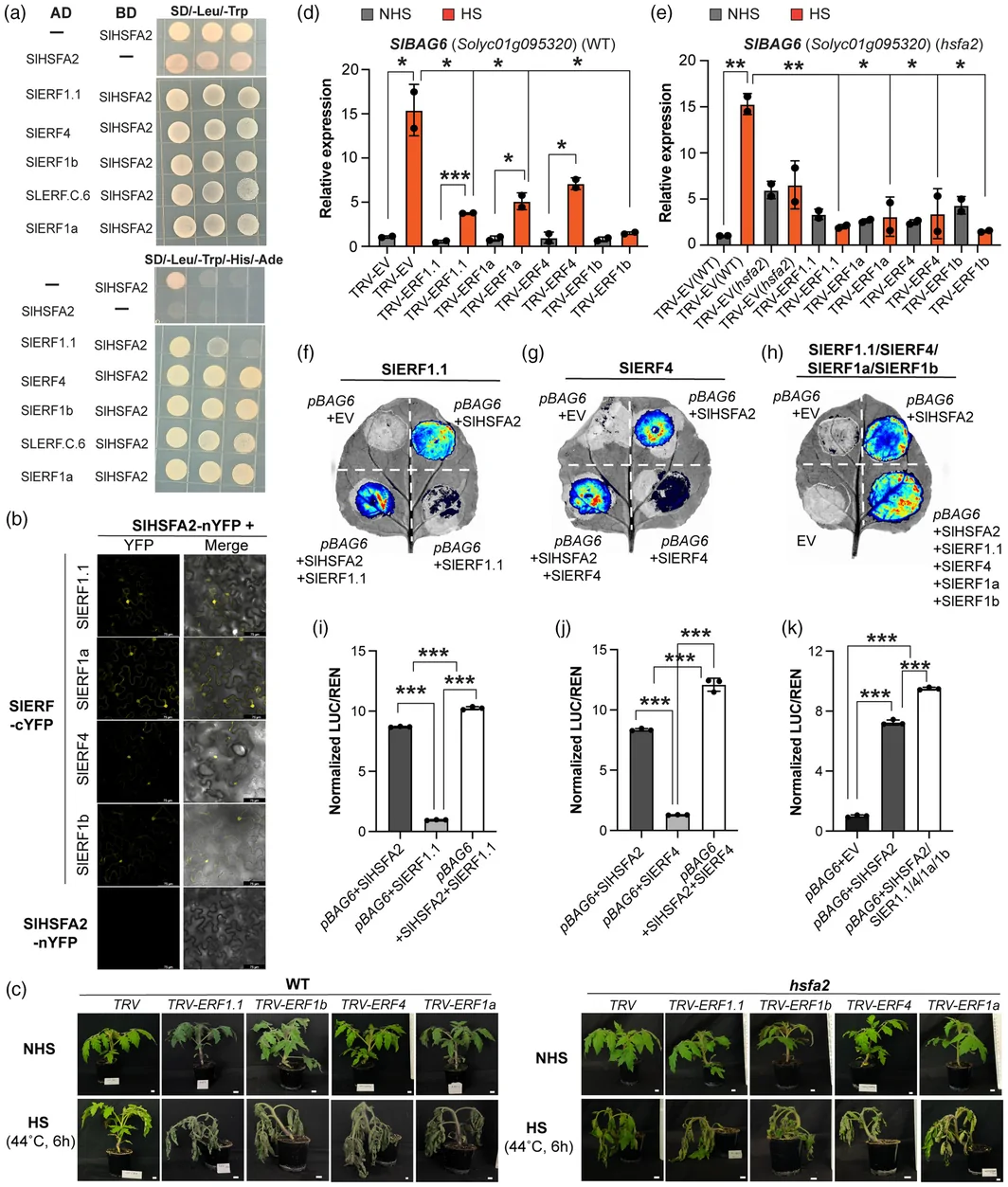
SlHSFA2 interacts with multiple ETHYLE RESPONSE FACTORs (SlERFs). (a) Yeast two‐hybrid (Y2H) assay showing interaction between SlHSFA2 and SlERFs *in vitro*. Yeast cells were grown on selective media (SD/‐Leu/‐Trp) and (SD/‐Leu/‐Trp/‐His/‐Ade). AD, GAL4 activation domain; BD, GAL4‐binding domain. (b) Bimolecular fluorescence complementation (BiFC) assay showing interaction between SlHSFA2 and SlERF1.1, SlERF1a, SlERF4, and SlERF1b at 37°C. The *N. benthamiana* leaves were co‐infiltrated with SlHSFA2 fused to the N‐terminal and SlERFs to C‐terminal halves of YFP. SlHSFA2‐nYFP was used as negative control. (c) Phenotype of non‐stressed (NHS) and heat‐treated *SlERF1.1‐, SlERF1b‐, SlERF4*‐, and *SlERF1a‐*silenced plants in wild‐type (WT) and *hsfa2* backgrounds. Images were taken directly after the treatment. (d, e) Expression level of *SlBAG6* in NHS and HS‐treated *SlERF1.1‐, SlERF1b‐, SlERF4*‐, and *SlERF1a‐*silenced plants in WT (d) and *hsfa2* (e) backgrounds. Error bars indicate SD (*n* = 2). (f–h) Luciferase reporter assay showing activation of *SlBAG6* promoter by SlHSFA2 and hyperactivation by SlHSFA2 with SlERF1.1. (f), SlERF4 (g) or all SlERFs (h) in 37°C in *N. benthamiana* leaves. (i–k) The quantifications of the luciferase activity presented in (d–h) as LUC/REN. Error bars indicate SD (*n* = 3). Statistical significance was calculated using Student's *t*‐test *P < 0.05; **P < 0.01; ***P < 0.001.

To investigate the functional relevance of these interactions, we silenced four *SlERFs* genes (*SlERF1.1, SlERF1b, SlERF4*, and *SlERF1a*) using a TRV‐based VIGS system in both WT and *hsfa2* mutant backgrounds (Figure 6c). Plants carrying the pTRV2‐EV were used as controls. Silencing efficiency was confirmed by the significant reduction of *ERFs* transcripts levels measured by qRT‐PCR (Figure S15A) and by the characterizing photobleaching phenotype observed in pTRV2‐*PDS* plants (Figure S15B). In addition, we quantified *pTRV2* transcript levels by qRT‐PCR and found no significant differences between pTRV2‐EV and pTRV2‐*ERF*‐silenced plants (Figure S15C,D), indicating comparable viral vector accumulation in those plants. To determine the specificity of gene silencing, we measured the expression of the remaining *SlERF* genes in each silenced line, including pTRV2‐EV control (Figure S15E). No significant changes were detected in the expression of non‐target *SlERF* genes, suggesting that the silencing was highly specific (Figure S15E).

Accordingly, under non‐stressed conditions, the *SlERF*‐silenced plants did not show any visible phenotypic changes compared with the pTRV2‐EV control plants. In contrast, after HS treatment (44°C) for 6 h pTRV2‐*SlERF1.1*, pTRV2‐*SlERF1a*, pTRV2‐*SlERF4*, and pTRV2‐*SlERF1b* plants exhibited strongly affected phenotypes after HS than the pTRV2‐EV (WT) control plants (Figure 6c; Figure S16). Silencing these *SlERFs* in the *hsfa2* mutant background resulted in phenotypes similar to those observed in corresponding WT *SlERFs*‐silenced plants, indicating that the loss of SlERF function produced a similarly strong reduction in thermotolerance independently of the genetic background. In contrast, EV‐*hsfa2* plants exhibited an intermediate phenotype, being more sensitive than pTRV2‐EV (WT) plants but less severely affected than ether *WT‐SlERF*‐silenced plants or *hsfa2 SlERF*‐silenced plants (Figure 6c; Figure S16). As expected, pTRV2‐EV (WT) plants showed no visible HS symptoms under non‐stressed conditions but displayed characteristic HS symptoms after 6 h at 44°C. Importantly, all p*TRV2‐SlERF*‐silenced plants, including p*TRV2‐ERF1.1*, p*TRV2‐ERF1b*, p*TRV2‐ERF4*, and p*TRV2‐ERF1a* exhibited reduced recovery after HS treatment compared with the pTRV2‐EV control (WT) (Figure 6c; Figure S16). These findings indicate that the targeted SlERF genes contribute to the full HS response and that their reduced expression compromises thermotolerance.

Next, we analyzed the expression of transcriptional thermal memory genes *SlBAG6*, for which we showed that SlHSFA2 acts as positive regulator of transcription, in *pTRV2‐SlERF*‐silenced plants in both WT and *hsfa2* backgrounds to assess whether SlERFs contribute to its regulation (Figure 6d,e). We found that *SlBAG6* expression was significantly reduced in *pTRV2‐SlERFs* lines in the WT background, whereas heat‐induced activation of *SlBAG6* was fully abolished in pTRV2‐SlERFs plants in the *hsfa2* mutant background (Figure 6d,e). This suggests that SlERFs contribute to the regulation of type II transcriptional thermal memory genes, including *SlBAG6*. This supports a model in which SlHSFA2 and SlERFs act in the same pathway during the HS to regulate the expression of transcriptional thermal memory genes type II.

Finally, we tested whether SlERFs activate *SlBAG6* promoter using a luciferase (LUC) reporter assay in *N. Benthamiana*. Effector constructs expressing SlHSFA2, SlERF1.1, and SlERF4 under the CaMV 35S promoter were co‐expressed with the *SlBAG6* promoter:LUC reporter (Figure 6f–h). Neither SlERF1.1 nor SlERF4 were able to activate the *SlBAG6* promoter when expressed alone (Figure S17). However, co‐expression of either together SlERF1.1 or SlERF4 with SlHSFA2 resulted in significantly higher *SlBAG6* promoter activity than activation of *SlBAG6* promoter by SlHSFA2 alone (Figure 6i,j). Similarly, co‐expression of all SlERF1.1, SlERF4, SlERF1a, and SlERF1b constructs together with SlHFA2 enhanced the *SlBAG6* promoter activity compared with SlHSFA2 alone (Figure 6k). These results suggest that SlERF proteins enhance SlHSFA2‐mediated activation of the *SlBAG6* promoter.

Together, these results support a role for SlERFs proteins as positive regulators of *SlBAG6* expression essential role in transcriptional thermal memory by co‐activating and potentially other transcriptional memory genes type II together with SlHSFA2.

## DISCUSSION

Transcriptomic thermal memory is an important adaptive strategy that enables plants to cope with sudden temperature fluctuations. This phenomenon has been well studied in the model plant Arabidopsis, where key molecular components of the HS memory network have been identified. However, the molecular mechanisms of transcriptional thermal memory in crops such as tomato remain poorly investigated, and it is unclear whether the underlying mechanisms are conserved. For instance, a recent study revealed that although barley thermal memory is dependent on conserved HSFs, the underlying molecular mechanisms differ from those reported in Arabidopsis (Pratx et al., 2025).

In this study, we investigated transcriptional thermal memory in tomato, one of the most widely consumed horticultural crops worldwide. Using the same thermal memory assay previously established for Arabidopsis (Olas & Wigge, 2025), we demonstrated that tomato seedlings are primable and exhibit transcriptional thermal memory capacity. This suggests that transcriptional thermal memory represents a conserved adaptive mechanism in plants, as Arabidopsis (*Brassicaceae*) and tomato (*Solanaceae*) belong to distinct plant families. Together with reports describing thermal memory in yeast (Berry & Gasch, 2008) and bacteria (Runde et al., 2014), our findings indicate that this phenomenon may be broadly conserved across kingdoms. It is important to note that although chromatin‐based regulation, protein stability, and other post‐transcriptional processes are known to contribute to thermal memory, this study specifically focused on the investigation of transcriptional thermal memory.

In Arabidopsis, the priming stimulus is remembered for 3–4 days (Sedaghatmehr et al., 2016; Stief et al., 2014). By contrast, we found that tomato seedlings (cv. *Moneymaker*) displayed a significantly longer memory phase. Effective priming was observed even after a 10‐day break between moderate priming stress and severe triggering stress. This prolonged memory duration is agriculturally relevant, as it provides extended natural protection. The longer memory phase in tomato may correlate with its extended vegetative phase compared with *Arabidopsis*, thereby ensuring meristem protection before flowering initiation occur to secure fruits production.

From a horticultural perspective, transcriptional thermal memory in tomato is highly relevant because tomato is particularly sensitive to temperature fluctuations (Cammarano et al., 2022; Peet et al., 1998; Pressman et al., 2002). HS during the reproductive stage impairs pollen viability and induces male sterility, ultimately reducing yield (Sato et al., 2000; Sato et al., 2002). Priming during the vegetative stage may therefore provide a natural form of plasticity that enables plants to adaptively respond to temperature fluctuations and safeguard productivity. This is especially important because maintaining optimal yield and fruit quality in tomato requires relatively stable optimal conditions (Van Ploeg & Heuvelink, 2005), which are increasingly difficult to maintain under current climate change scenarios. In fact, tomato production has been predicted to decrease by ~6% by 2050 only due to rising global temperatures (Cammarano et al., 2022). Therefore, developing strategies that reduce the heat sensitivity during the reproductive stage is essential.

Interestingly, P + T plants produce more fruits than control NHS and primed‐only plants under controlled conditions as well as under field conditions, although NHS and P + T plants produce a similar number of flowers. Interestingly, increased yield was not observed in primed‐only plants, suggesting that thermal priming alone is insufficient to enhance yield and that its beneficial effects become evident only upon exposure to both priming and triggering treatments. Consistent with this observation, thermal priming did not promote shoot growth in tomato plants. In contrast, enhanced shoot growth was observed after subsequent triggering stress, supporting the idea that priming improves recovery from recurrent stress rather than stimulates growth. Together, these findings suggest that although transcriptional thermal memory is transient, its developmental and physiological consequences can persist beyond the memory phase and positively influence subsequent plant performance, including reproductive development and fruit yield. This further supports the notion that activation of heat‐responsive genes during priming and triggering may influence not only stress adaptation but also developmental pathways that contribute to improved growth; however, the mechanisms linking thermal memory in tomato seedlings to increased yield remain to be determined (Charng & Chen, 2023; Fragkostefanakis et al., 2016).

Recent studies have shown that developmental processes such as shoot branching are regulated in a temperature‐dependent manner through SlTCP15 (Debnath & Olas, 2025a; Song et al., 2025). In tomato, heat‐responsive TFs such as SlHSFA2 are required for pollen thermotolerance during microsporogenesis by activating the expression of *SlHSPs* in developing anthers and pollens (Fragkostefanakis et al., 2016), while overexpression of SlHSFA2 promotes callus growth (Ogawa et al., 2007). Several studies have also highlighted the role of the shoot apical meristem in HS adaptation. In Arabidopsis, *CLV1* and *CLV3* expression is transiently suppressed during priming and triggering but returns to control levels after HS (Olas et al., 2021), similar to what we observed in tomato. In addition, WUS contributes to thermotolerance by directly controlling the expression of genes involved protein folding (Debnath & Olas, 2025b; Liu et al., 2024). Consistent with this, HS disrupts SlWUS homeostasis in tomato, leading to an increased proportion of malformed fruits (Wu et al., 2024). Finally, Lu et al. (2025) showed that *THERMOSENSITIVE PARTHENOCARPY 4a* and *4b* play essential roles in maintaining fruit set under HS in tomato, whereas Sun et al. (2024) demonstrated that *qMULTIPLE INFLORESCENCE BRANCH 2* modulates inflorescence branching in response to elevated temperature.

We identified SlHSFA2 as a component of the transcriptional thermal memory response in tomato, similar to what has been reported in Arabidopsis (Charng et al., 2007; Friedrich et al., 2021; Oberkofler et al., 2025). The *hsfa2* mutant lacked the capacity to retain the priming stimulus, demonstrating that SlHSFA2 is required for memory maintenance. Although previous studies suggested that SlHSFA2 acts as co‐activator of SlHSFA1a in response to elevated temperature (Scharf et al., 1998), our results show that during recurrent HS, SlHSFA2 alone can activate the promoter of the type II transcriptional memory gene – *SlBAG6*. This regulatory role may extend to additional transcriptional memory genes, as we found that majority of upregulated type II transcriptional thermal memory genes contain HSE motifs in their promoter regions and that their expression is downregulated in P + T *hsfa2* mutants, suggesting involvement of SlHSFA2 in their regulation. However, this would need to be tested individually for each gene. It is important to note that TRV‐*SlBAG6*‐silenced plants showed a more severe phenotype than TRV‐*hsfa2* plants. To exclude nonspecific effects of the VIGS construct, we assessed it silencing specificity. Our data suggest that SlHSFA2 represents only one component of the HS regulatory network controlling *SlBAG6* expression. Consistent with this model, we found that heat‐induced *SlBAG6* expression was only reduced in TRV‐*hsfa2* plants and not completely abolished, indicating that additional heat‐responsive TFs, including other HSFs may also contribute to its regulation. Therefore, the stronger phenotype observed in TRV‐*SlBAG6*‐silenced plants is likely explained by the fact that *SlBAG6* functions downstream of multiple regulatory pathways. It is also important to note that the expression analyses in the VIGS experiment were performed using leaf tissue, whereas the expression of *SlBAG6* in P + T *hsfa2* mutant was analyzed in shoot apices. In the P + T *hsfa2* mutant, heat‐induced *SlBAG6* expression was fully abolished in shoot apices, suggesting that SlHSFA2 might act as the predominant regulator of *SlBAG6* at the SAM. Thus, the partial induction of *SlBAG6* in leaves of TRV‐*hsfa2* suggest a presence of tissue‐specific regulation, rather than a lack of dependence on SlHSFA2. In fact, we previously showed that Arabidopsis SAM displays an autonomous thermal memory that involves a different set of TFs and target genes (Olas et al., 2021). Furthermore, additional studies are required to investigate the chromatin dynamics at *SlBAG6* locus and determine whether SlHSFA2 regulates *SlBAG6* solely at the transcriptional level or also through chromatin‐mediated mechanisms.

We also showed that some transcriptional thermal memory genes such as *SlERF1.1*, which lack HSE motifs in their promoters, can also be regulated independently of SlHSFA, although their expression was significantly reduced in P + T *hsfa2* mutants compared with P + T control plants. This suggests the involvement of additional, yet unidentified, TFs that contribute to the regulation of transcriptomic thermal memory in tomato (Figure 7).

**Figure 7:**
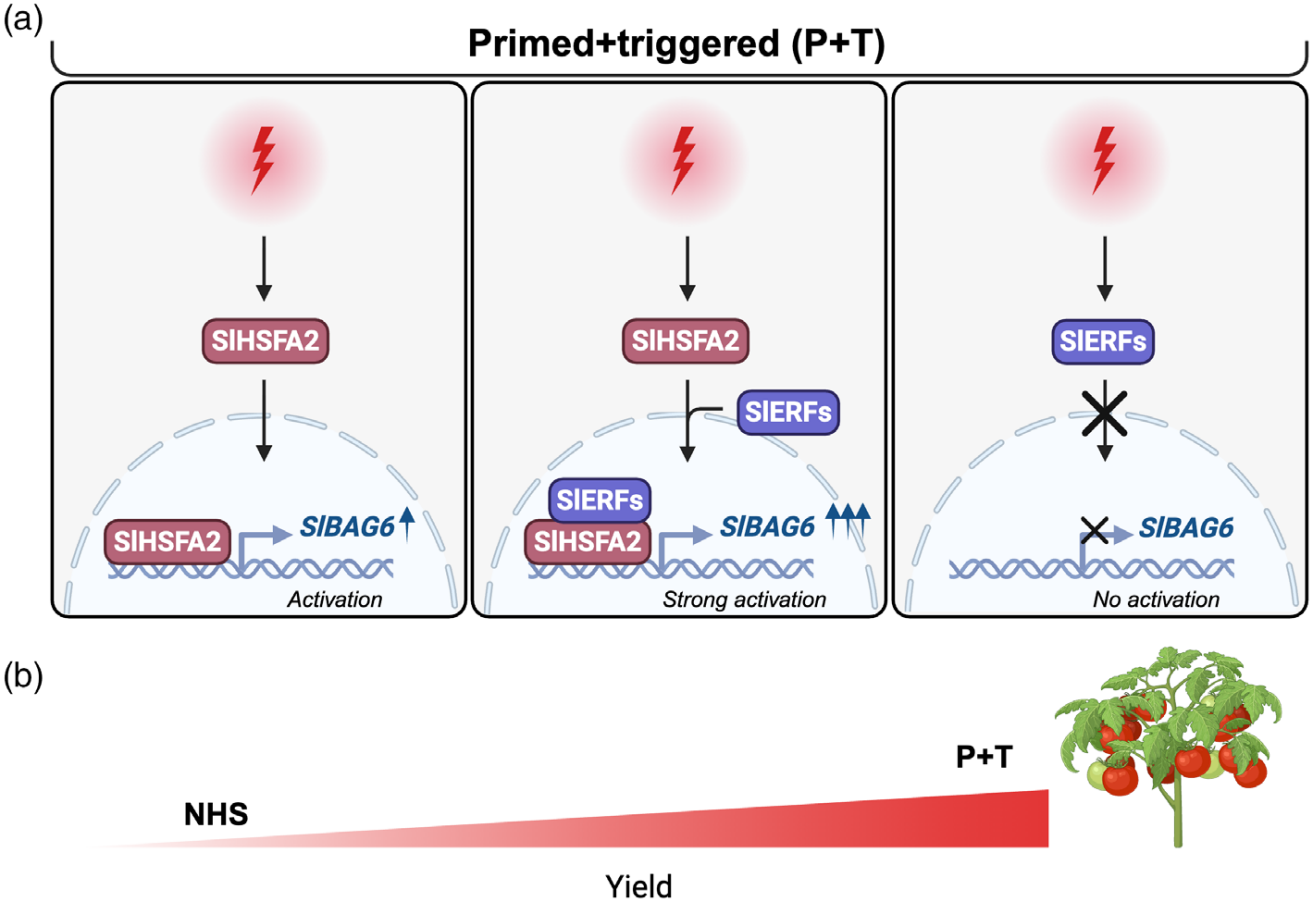
Simplified model illustrating the regulation of transcriptomic thermal memory in tomato plants. (a) In previously primed plants exposed to triggering stress (primed + triggered, P + T), transcriptional thermal memory genes containing heat shock elements (HSE) in their promoter regions, including *SlBAG6*, are activated by SlHSFA2. In addition, the interaction between SlHSFA2 and ETHYLENE‐RESPONSIVE FACTORs (SlERFs) enhances the activation of *SlBAG6*. Notably, SIERFs alone do not activate the *SlBAG6* promoter. (b) Thermal priming after subsequent stress results in a significant increase in yield in P + T under both controlled and field conditions compared with non‐heat‐stressed (NHS) plants.

Interestingly, we found that several SlERFs contribute to regulation of type II transcriptional thermal memory genes by forming a protein complex with SlHSFA2. The physical interaction between SlHSFA2 and SlERF1.1, SlERF1a, SlERF1b, and SlERF4, together with the enhanced heat sensitivity of the corresponding SlERF‐silenced plants, supports the existence of an SlHSFA2–SlERF regulatory module that promotes thermotolerance in tomato. Silencing of these SlERF resulted in similarly severe phenotypes in both WT and *hsfa2* backgrounds, whereas EV‐*hsfa2* plants displayed an intermediate phenotype. This absence of an additive effect suggests that SlHSFA2 and the SlERFs function within the same or converging regulatory pathways. However, considering that SlERF silencing caused a more severe phenotype than loss of SlHSFA2 alone, these SlERFs are unlikely to function exclusively with SlHSFA2 and are instead likely to cooperate with additional HSFs during HS response.

This interpretation is further supported by the expression analyses of *SlBAG6*, a common gene whose promoter is activated by SlHSFA2 and whose activation is further enhanced by the presence of SlERFs. Although *SlBAG6* transcript levels were reduced in the *hsfa2*‐TRV mutant, heat‐induced activation was not fully abolished, suggesting that SlHSFA2 is an important but not exclusive, regulator of *SlBAG6* expression. Interestingly, *SlBAG6* expression was reduced in *SlERF*‐silenced WT plants after HS, although *SlBAG6* transcript levels remained higher in those plants compared with the corresponding *SlERF*‐silenced plants under non‐stress conditions, indicating that heat‐induced *SlBAG6* expression is largely dependent on both SlHSFA2 and SlERFs. We also showed that SlHSFA2 alone can activate *SlBAG6* promoter, while SlERFs alone were unable to do this. Co‐expression of both SlHSFA2 and SlERFs significantly enhanced *SlBAG6* promoter activity, suggesting that SlERFs act as co‐transcriptional activators, potentially interacting with others HSFs to control *SlBAG6* expression. Further suggesting the existence of a complex regulatory network underlaying transcriptional thermal memory and thermotolerance in tomato that involves interactions between SlERFs and SlHSFs.

This hypothesis is consistent with previous studies showing extensive crosstalk between HSFs and ERFs. For instance, in Arabidopsis ERFs have been shown to directly bind to the promoter of HSFA2 to enhance thermotolerance (Huang et al., 2021), while HSFA7 has been shown to directly control expression of genes involved in ethylene synthesis and signaling (John et al., 2024), highlighting a complex regulatory network between ERFs and HSFs.

Together, our results show that tomato plants possess transcriptional thermal memory and that duration is considerably longer than that reported for Arabidopsis. Thermal priming following recurrent triggering improves fruit set both under controlled and field conditions, without compromising fruit quality, pointing to a novel strategy for improving yield under fluctuating temperature conditions (Figure 7). We further identify SlHSFA2 and SlERFs as components of the transcriptional thermal memory network in tomato. Although their role in increasing yield remains to be investigated by assessing the yield of overexpression and CRISPR‐generated lines. These findings provide a foundation for developing heat‐resilient tomato plants and offer new opportunities for breeding strategies aimed at improving crop performance under climate change.

## MATERIAL AND METHODS

### Plant material and growth conditions

*Solanum lycopersicum cv. Moneymaker* was used as the WT plant. Seeds were placed on 0.5 × MS medium with 1% sucrose, stratified at 4°C (dark) for 2 days, and transferred to a controlled growth chamber (Polyklima HM4Z‐TDLPlus‐FarRed, polyklima® GmbH, Germany) under long‐day conditions (LD, 16 h light/8 h darkness) at 22°C. Thermal priming assays were performed in a water bath as descried previously (Olas & Wigge, 2025). 5‐day‐old seedings were subjected to a priming treatment (moderate stress, 37°C for 1.5 h, 1.5 h; 22°C for 1.5 h, followed by 44°C for 45 min). Plates were returned to the growth chamber for 3, 5 or 10 days (memory phase) before triggering stress (44°C for 1.5 h). Survival phenotypes were classified as previously reported for Arabidopsis: green, weakly damaged, severely damaged, and dead. The *hsfa2* mutant was described previously (Hu et al., 2020).

### Virus‐induced gene silencing VIGS

VIGS constructs were generated using the pTRV1/pTRV2 VIGS system (Liu et al., 2002). The primers were designed using the solgenome VIGS tool (online software) for the genes *SlPDS*, *SlERF1.1*, *SlERF1a*, *SlERF1b, SlERF4*, and *SlBAG6*. The 500 bp fragments were amplified using gene‐specific primers and subsequently cloned into the TRV2 vector.

*Agrobacterium* strain *GV3101* was transformed with the TRV2 constructs. VIGS assay was performed as described previously (Kumar et al., 2015; Liscombe & O'Connor, 2011) in WT and *hsfa2* mutant backgrounds. Plants were subjected to 6 h HS (44°C) in the incubators.

### Yield analyses

For controlled conditions, control NHS and P + T WT plants were transferred from plates to soil (pots) 3 days after triggering (DAT; 13 days after germination (DAG)) and grown in a controlled walk‐in growth chamber (Regineering GmbH, Germany) under LD conditions at 22°C. Flowering time was recorded, and total fruit number per plant was determined at 65 days after first flower opened. Total fruit weight, average mature fruit weight, mature fruit area, and seed number were measured. Experiment was performed twice with each replicate containing >5 plants per treatment.

For field experiment, NHS and P + T plants were transferred to soil (pots) at 3 DAT (13 DAG) and kept in controlled growth conditions. Plants were then transferred to an open greenhouse at 10 DAT (20 DAG) for acclimatization, and at 17 DAT (27 DAG) transferred to the field. Yield (total fruit number) was scored 60 days after the first flower appeared. Field experiment was conducted from June to September 2025 in Großbeeren (Brandenburg, Germany).

### Analysis of carotenoids

Freeze‐dried material was pre‐ground in 20 mL tubes (Retsch mill) and then ground three times in cooled 2 mL Eppendorf tubes. Extraction was carried out as described (Frede et al., 2019) with a few modifications. Briefly, 8 mg of material was extracted 2× with 750 μL tetrahydrofuran/methanol (1:1; 5 min, 20°C, 1450 rpm), followed by 500 μL chloroform (stabilized with ethanol). Supernatants were combined, dried under nitrogen stream, redissolved in 50 μL dichloromethane and 200 μL isopropanol (Merck, Darmstadt, Germany) with 2 min ultrasonication, centrifuged (4500 rpm, 5 min), filtered (0.22 μm PTFE filters), and stored at 10°C.

Carotenoids were separated on a YMC C30‐chromatographic column (100 × 2.1 mm, 3 μm, YMC Co. Ltd., Kyoto, Japan) using a 1290 Infinity II UHPLC with diode array detection, coupled to a 6230 TOF LC/MS ESI mass spectrometer (Agilent Technologies, Waldbronn, Germany). The injection volume used was 2 μL, the column temperature was 20°C, and the flow rate was 0.200 mL/min. The mobile phase was composed of (A) methanol/water (96:4, v/v, containing 20 mM ammonium acetate) and (B) tert‐butylmethylether/methanol/water (90:6:4, v/v/v, also containing 20 mM ammonium acetate). The gradient elution was employed as follows: 0% of B from 0 to 10 min, then from 0 to 20% of B from 10 to 17 min, keeping at 20% of B from 17 to 45 min, then from 20 to 100% of B from 45 to 55 min, keeping at 100% of B from 55 to 70 min, and returning to 0% of B from 70 to 71 min. Additional 10 min was used for column re‐equilibration. β‐carotene and lutein were quantified by external calibration with authentic standards (λ = 450 nm).

### RNA‐seq

Total RNA was extracted from shoot apices of WT and *hsfa2* tomato plants grown in LD conditions harvested after priming and triggering treatments in three biological replicates. Samples were harvested at 0.5 h and 2 h after priming and 0.5 h after triggering stress, with corresponding non‐heat‐stressed (NHS) controls. A total of 70 mg of dissected plant material was homogenized to powder using Qiagen TissueLyser II (Qiagen). Total RNA was extracted using RNeasy Plant Mini Kit (Qiagen) according to the manufacturer's instructions. Total RNA was quantified using Qubit4 (Thermo Fisher Scientific) according to the manufacturer's instructions and tested for integrity using an RNA screen tape in an Agilent2200 tape station (Agilent). 1 ug ultrapure total RNA from WT and *hsfa2* genotypes was used to prepare high‐throughput mRNA sequencing libraries using TruSeq. Stranded mRNA Library Prep (Illumina, 20 020 595) and sequenced on a *NextSeq*500 (Illumina) according to manufactures instructions.

The *Solanum lycopersicum* reference genome ITAG4.0 and corresponding gene annotation were obtained from the Sol Genomics Network (Fernandez‐Pozo et al., 2015). Gene annotations were converted from GFF3 to GTF format using gffread from Cufflinks v2.2.1 (Trapnell et al., 2010) to ensure the presence of “gene_id” and “transcript_id” attributes required for downstream analyses. Adapter trimming and quality filtering were performed with fastp v0.23.4 (Charng & Chen, 2023) using Illumina TruSeq paired‐end adapter sequences with the following options: length_required = 36, correction, cut_right, cut_right_window_size = 4, cut_right_mean_quality = 15, trim_poly_g, trim_front1 = 3, and trim_tail1 = 3. Read quality was assessed with FastQC v0.12.1 (Simon Andrews et al., 2010). Trimmed paired‐end reads were aligned to the tomato reference genome using STAR v2.7.11b (Dobin & Gingeras, 2015) with the following non‐default parameters: alignIntronMax = 12 000, outFilterMismatchNmax = 999, outFilterScoreMinOverLread = 0.3, outFilterMatchNminOverLread = 0.3. Aligned reads were quantified using HTSeq 2.0.5 (Putri et al., 2022). Genes with zero counts across all samples were excluded prior to downstream analyses. Differential gene expression analysis was performed with DESeq2 v1.38.3 (Love et al., 2014). DEGs were as genes with an adjusted *P*‐value (false discovery rate, FDR) <0.05. The pairwise group comparisons tested for differential expression were “WT_10C” versus “WT_10PT,” “hsfa2_10C” versus “hsfa2_10PT,” and “WT_10PT” versus “hsfa2_10PT.” For each comparison, DESeq2‐normalized counts were extracted from the DESeq2 object and used to compute group‐wise mean expression and standard deviation for control and treated samples. Heatmaps were generated from variance‐stabilized expression values obtained using the DESeq2 variance stabilizing transformation. Gene ontology (GO) enrichment analysis was performed using gprofiler2 v0.2.3 (Kolberg et al., 2020).

### Quantitative reverse transcription PCR (qRT‐PCR)

Total RNA from shoot apices of WT and *hsfa2* mutants and leaves of after pTRV2‐silenced plants in WT and *hsfa2* mutant background plants was isolated using RNA easy kit (Qiagen). cDNA was synthesized using LunaScript RT SuperMix Kit (New England Biolabs, USA) according to the manufacturer's instructions. Reactions were performed on a LightCycler480 II (Roche, Swiss) using SYBR Green‐PCR Master Mix (Applied Biosystems/Life Technologies, Germany). *ACTIN* (*Solyc03g078400*) was used as the housekeeping gene. Ct values were normalized to the housekeeping gene and presented as fold change, as previously described (Olas et al., 2019). Primers are provided in Table S8.

### RNA *in situ* hybridization

Tomato meristems from WT and mutant plants were processed and fixed using HistoCore PEARL system (Leica, Wetzlar, Germany), embedded in paraffin wax using HistoCore Arcadia H (Leica, Wetzlar, Germany), and sectioned using a rotary microtome SN4150 (Leica, Wetzlar, Germany). Probes were generated using the SP6/T7 transcription kit (Roche, Germany). Primers are provided in Table S8. RNA *in situ* hybridization was performed as previously described for *Arabidopsis* (Olas et al., 2019).

### Trypan blue staining

Trypan blue staining was performed as previously described (Fernández‐Bautista et al., 2016). Twenty‐four hours after triggering treatment, seedlings of WT control, primed, P + T, and T‐only plants were incubated in 0.4 mg/mL Trypan Blue dissolved in phenol/glycerol/lactic acid/water/ethanol (1:2:1:1:8) for 30 min at 22°C, and subsequently washed 3× with 90% ethanol.

### Luciferase reporter assay

A 901 bp *SlBAG6* promoter fragment was amplified from the WT genomic DNA and cloned into the pGreen800‐LUC vector at the *BamHI* site using SLIC‐based cloning to generate the reporter construct. Effector constructs were generated by cloning the CDS of *SlHSFA2*, *SlERF1.1*, *SlERF1a*, *SlERF1b*, and *SlERF4* into the pJHA212K_35S_3xN‐FLAG_SLIC vector at the *BamHI* site under CaMV 35S promoter. All constructs, including empty vectors, were transformed into *Agrobacterium* strain *GV3101*. For the promoter construct, the pSoup helper plasmid was also transformed. *Agrobacterium* cultures were diluted to OD600 = 0.5 with infiltration buffer (1/2 MS (pH 5.7), 1% sucrose, 10 mM MgCl_2_.6H_2_O, 10 mM MES, 0.005% (v/v) Silwet L‐77, 100 μM acetosyringone) and infiltrated to fully expanded *N. benthamiana* leaves at a 1:1 ratio. For the paired luciferase assay in which all SlERFs were co‐infiltrated with SlHSFA2 to test their combined effect on the *SlBAG6* promoter, constructs were mixed at a 1:1:1:1:1:1 ratio. For comparison with SlHSFA2 alone, the same ratio was maintained by supplementing with EV effector constructs. A mixture of both empty vectors was used as a negative control. After overnight incubation in the dark, plants were kept at 22°C for 24 h. Before imaging, leaves were infiltrated with 1 mM D‐luciferin and imaged using a BioRad imaging system. Relative luminescence was quantified using the Promega Dual‐Luciferase Reporter Assay System. The images shown are representative of three independent biological replicates. Primers are provided in Table S8.

### Yeast two‐hybrid assay

The coding sequences of the *SlHSFA2* were cloned into the pGBKT7 vector (containing the GAL4‐binding domain) using HiFi DNA Assembly Master Mix (New England Biolabs, USA). For the prey constructs, the coding sequences of *SlERF1.1*, *SlERF1a*, *SlERF.C.6, SlERF1.2*, *SlERF1b, SlERF4*, and *AP2‐like ethylene‐responsive transcription factor* were cloned into the pGADT7 vector (containing the GAL4 activation domain). Positive clones were transformed into the yeast strain AH109. To test protein–protein interactions, yeast cells harboring BD‐HSFA2 and activation domain (AD)‐ERFs were mated. The empty GAL4 BD bait vector (pGBKT7) and GAL4 AD prey vector (pGADT7) were used as negative controls. Three days after mating, the cells were plated on selection media: SD–Leu–Trp (mating control) and SD–Leu–Trp–His–Ade to assess positive interactions. Primers used are provided in Table S8.

### Colocalization study in *Nicotiana benthamiana*

The CDS of *SlERF1.1*, *SlERF.C.6, SlERF1a*, *SlERF1.2*, *SlERF1b, SlERF4*, and *AP2‐like ethylene‐responsive transcription factor* were cloned into pCHF3 vector in fusion with an *mVenus* reporter at the C‐terminus under the 35S promoter. *SlHSFA2* was cloned into the *pCHF3* vector in fusion with an *mTurquoise* reporter at the C‐terminus under the 35S promoter. Overnight cultures of *Agrobacterium* strain *GV3101* harboring the *35S: ERFs _ mVenus* and *35S: HSFA2_ mTurquoise* constructs were resuspended in infiltration buffer (1/2 MS (pH 5.7), 1% sucrose, 10 mM MgCl_2_•6H_2_O, 10 mM MES, 0.005% (v/v) Silwet L‐77, 1/1000 acetosyringone from 100 μM stock). Constructs were mixed 1:1 (total OD600 = 0.8) and infiltrated into the abaxial sides of ~20‐day‐old *N. benthamiana* leaves. After overnight incubation (dark), plants were transferred to light at 22°C for 1 day. Imaging was performed with a confocal microscope using sequential mVenus (excitation 515 nm, emission 527 nm) and *mTurquoise* (excitation 434 nm, emission 474 nm) channels. Images shown are representative of at least 10 independent measurements revealing similar results.

### Bimolecular fluorescence complementation (BiFC)

The CDS of *SlHSFA2* and *SlERF.C.6, SlERF1.1*, *SlERF1a*, *SlERF1.2*, *SlERF1b, SlERF4, AP2‐like ethylene‐responsive transcription factor* were amplified from WT plant cDNA using primers designed for SLIC‐based cloning and cloned into *pUCL3L2* using SalI and PstI sites. *SlHSFA2* was cloned into the entry vector *pUCL1L4* using XbaI and PstI restriction sites. Positive clones were confirmed by sequencing and subsequently used in an LR reaction with destination vector *pBiFCt‐2in1‐CC*, which conatins both YFP halves (C‐terminal nYFP and cYFP). For negative controls, pBiFCt‐2in1‐CC vectors containing only *SlHSFA2*‐*cYFP* and empty nYFP cassettes were used. The pBiFCt‐2in1‐CC plasmids containing *SlHSFA2* and/or *SlERFs* were transformed into the *Agrobacterium* strain *GV3101* and infiltrated into *N. benthamiana* leaves. Plants were imaged using a Leica confocal microscope with the Alexa 514 channel (excitation 513 nm, emission 530 nm). The images shown are representative of at least 10 independent measurements showing similar results.

### Statistical analysis

A two‐tailed Student's *t*‐test was used to analyze the statistical significance difference compared with control conditions (*P < 0.05; **P < 0.01; ***P < 0.001), unless indicated otherwise.

## Author Contributions

PD performed most of the experiments. KK conducted the BiFC and assisted with the VIGS experiments. PD, KK, and CA carried out yield experiments. PK performed all RNA‐seq analyses. KJ carried out RNA‐seq experiments using material provided by PD, PB measured metabolites. VH took care of weather station. NMvD supervised the work from PB. PW supervised the work of KJ. JJO conceived and supervised the study and wrote the manuscript. All authors agreed with the submission of the final manuscript.

## Conflict of Interest

The authors declare no competing interests.

## Supporting information


**Figure S1.** Thermopriming phenotype of tomato wild‐type plants with different memory lengths grown under long‐day photoperiod (16 h light and 8 h darkness). (A) Thermopriming phenotype of non‐stressed control (NHS), primed (P), primed + triggered (P + T), and triggered (T) tomato plants after a memory phase of 3 days. (B) Survival rate of WT plants in panel (A) and (C–E). (C–E) Thermopriming phenotype of non‐stressed control (NHS), primed (P), primed + triggered (P + T), and triggered (T) tomato plants after a memory phase of (C) 10 days, (D) 20 days, or (E) 25 days. (F, G) Shoot fresh weight, and shoot length of NHS, P, P + T, and T tomato plants with 5‐day memory phase. Error bars represent SD (*n* = 5, except for T‐only plants where *n* = 4). Statistical significance was calculated using a student's *t*‐test *P < 0.05; **P < 0.01; ***P < 0.001. (H) Trypan blue stationing of NHS, P, P + T, and T tomato seedlings with a 5‐day memory phase performed 24 h after triggering stress.
**Figure S2.** Detailed phenotypic characterization of control (NHS), primed‐only (P), primed + triggered (P + T) tomato wild‐type plants grown in long day (16 h light and 8 h darkness) under controlled conditions and exposed to thermopriming treatment with a 5‐day memory phase. (A) qRT‐PCR analysis showing the expression of *CLAVATA1* (*SlCLV1*) in meristems of NHS and primed plants at 0.5 and 2 h after priming, and in meristems of NHS, P + T, and T‐only plants at 0.5 and 6 h after triggering. Error bars represent SD (*n* = 2). Statistical significance was determined using a student's *t*‐test *P < 0.05; **P < 0.01; ns (not significant). (B, C) Yield phenotype of NHS and P + T plants from a second independent experiment, assessed 60 days after first flower appearance under controlled growth conditions. (D) Number of fruits produced by NHS and P + T plants at 60 days after the appearance of the first flower from a second independent experiment. (E) Total fruit weight of fruits produced by NHS and P + T plants grown in controlled growth chamber from a second independent experiment. Error bars in (D, E) represent SD (*n* = 6 for NHS, and *n* = 5 for P + T). (F) Ripening phenotype of NHS and P + T fruits. (G) Plant height of 1‐month‐old NHS, P‐only, and P + T tomato plants grown under controlled conditions. (*n* = 5). (H) Phenotypes of 1‐month‐old NHS, P‐only, and P + T plants grown under controlled conditions. Statistical significance was calculated using a student's *t*‐test *P < 0.05; **P < 0.01; ***P < 0.001.
**Figure S3.** The average air temperature recorded during field experiment (from July till September) using the weather station collocated at the field.
**Figure S4.** Phenotype of control (NHS) and primed + triggered (P + T) tomato wild‐type plants with a 5‐day memory phase grown under non‐controlled conditions (field experiment). Priming was performed at 5 days after germination (DAG) and triggering treatment at 10 DAG. Plants were transferred to small pots at 13 DAG (3 days after triggering; DAT), moved to an open greenhouse for acclimatization at 20 DAG (10 DAT), and finally transplanted to the field at 27 DAG (17 DAT). Experiment was performed once.
**Figure S5.** Phenotypic characterization of non‐stressed control (NHS) and primed + triggered wild‐type tomato plants grown under non‐stressed conditions (field experiment). (A) Plant height of NHS and P + T plants over time in the field experiment. (B) Root phenotype of NHS and P + T plants on the day of transfer to soil (field); 27 days after germination (DAG)/20 DAT (days after triggering). (C, D) Root fresh weight (C) and dry weight (D) of NHS and P + T plants analyzed at 27 DAG/20 DAT. (E) Diameter of mature red fruits. (F, G) Number of seeds produced by NHS and P + T plants. Error bars represent SD. Statistical significance was calculated using a student's *t*‐test *P < 0.05, ***P < 0.001.
**Figure S6.** RNA‐seq analysis of control (NHS), primed (P), primed + triggered (P + T), and triggered (T) wild‐type tomato plants with a 5‐day memory phase. (A) Principal component analysis (PCA) of TPM values showing clustering of meristem samples of NHS (gray), P (orange), P + T (red), and T‐only (black) plants during the thermomemory. The 5NHS samples serve as controls for the 5P samples, whereas the 10NHS samples correspond to the P + T and T samples. (B) Heatmap of TPM values showing the average correlation among all samples. (C, D) Expression level of *HEAT SHOCK PROTEIN 17.6* (*SlHSP17.6*) and *HEAT SHOCK FACTOR A2* (*SlHSFA2*) analyzed at the shoot apex of NHS and P‐only plants at 0.5, 2, 24, 72, and 120 h after priming. Error bars represent SD. Statistical significance was calculated using a student's *t*‐test ***P < 0.001.
**Figure S7.** Transcriptional thermal memory genes. (A) Venn diagram of significantly upregulated DEGs (p_BH <0.05) in *P* versus NHS at 0.5 h after priming and in P + T versus NHS at 0.5 h after triggering. (B) Venn diagram of significantly upregulated DEGs (p_BH <0.05; Log2FC ≥1) in *P* versus NHS at 0.5 h after priming and in P + T versus NHS at 0.5 h after triggering. (C) Heatmap showing the expression of differentially expressed *HEAT SHOCK FACTORs* (*HSF*s) genes after priming and triggering, presented as Log2 fold change (log2 FC). (D) Expression level of thermal memory genes analyzed with *Clathrin Adaptor Complexes medium subunit (SlCAC)* as housekeeping gene. Error bars represent SD. Statistical significance was calculated using a student's *t*‐test **P < 0.01, ***P < 0.001.
**Figure S8.** Relative expression of *WUSCHEL* (*SlWUS*) analyzed at the shoot apex of NHS and P + T WT and *hsfa2* mutant plants at 0.5 h after triggering. Error bars indicate SD (*n* = 3). Statistical significance was calculated using Student's *t*‐test *P < 0.05; **P < 0.01.
**Figure S9.** RNA‐seq analysis of non‐heated (NHS) and primed + triggered (P + T) WT and *hsfa2* mutant plants at 0.5 h after triggering. (A) Principal component analysis (PCA) of TPM values showing clustering of meristem samples of NHS (C, gray), and P + T (PT, red) plants at 0.5 h after triggering. The 10C samples serve as controls for the 10PT samples. (B, C) Number of differentially expressed genes (DEGs) in the shoot apex of NHS and P + T WT and *hsfa2* mutant plants with log_2_FC ≥ |1| (B) or log_2_FC ≥ |2| (C).
**Figure S10.** Heatmap of genes common between transcriptional upregulated thermal memory genes (267 genes) and differentially expressed genes (2825 genes; Log2FC ≥1, pBH <0.05) in *hsfa2* NHS vs. *hsfa2* P + T 0.5 h after triggering, based on VST values. Note that nine genes did not show a transcriptional memory pattern in second independent RNA‐seq experiment and were therefore removed from the heatmap resulting in 83 genes. First, mean values per group were calculated, and then z‐scores were computed per gene.
**Figure S11.** Expression level of memory genes. Expression level of *B‐CELL LYMPHOMA 2* (*Bcl‐2*)‐*ASSOCIATED ATHANOGENE 5* (*SlBAG5*), *HEAT SHOCK PROTEIN 17.6* (*SlHSP17.6*) and *ETHYLENE‐RESPONSIVE FACTOR 1.1*. (*SlERF1.1*) at the shoot apex of control, not‐stressed (NHS) and primed + triggered (P + T) wild‐type (WT) and *hsfa2* mutant plants. Error bars represent SD (*n* = 3). Statistical significance was calculated using a student's *t*‐test *P < 0.05.
**Figure S12.** Luciferase reporter assay and quantifications showing activation of *SlERF1.1* promoter by SlHSFA2 in 37°C in *N. benthamiana* leaves. The quantification is presented as LUC/REN. Error bars represent SD (*n* = 6). Statistical significance was calculated using a student's *t*‐test ns, not significant.
**Figure S13.** Heatmap showing the expression of ethylene‐related genes with log_2_ fold change (log_2_FC) >3 in primed + triggered (P + T) wild‐type (WT) tomato and *hsfa2* mutant plants after triggering stress. Expression values were calculated from RNA‐seq data and are shown as log_2_FC relative to non‐stress controls (NHS). Red color indicted upregulation and blue downregulation.
**Figure S14.** SlERFs and SlHSFA2 colocalization and interaction. (A) Colocalization of *35S::HSFA2‐mTurquoise* and various *35S::ERFs‐mVenus* in *N. benthamiana* leaves at 37°C. (B) Yeast two‐hybrid (Y2H) assay showing SlERFs fused to the GAL4 activation domain co‐transformed with the empty GAL4‐binding domain (BD) vector. Yeast cells were grown on selective media (SD/‐Leu/‐Trp) and (SD/‐Leu/‐Trp/‐His/‐Ade). (C) Bimolecular fluorescence complementation (BiFC) assay showing interaction between SlHSFA2 and SlERF.C6., SlERF1.2, SlAP2‐like factor, and SlMBF1‐ER24 at 37°C in *N. benthamiana* leaves. Note that constructs expressing SlHSFA2 fused to the N‐terminal half of YFP and other protein fused to C‐terminal half of YFP.
**Figure S15.** SlERFs‐silenced plants. (A) Relative transcript level of *ETHYLENE‐RESPONSIVE FACTORS* (*SlERFs*) measured in ERFs‐silenced and TRV‐EV control plants exposed to heat stress (HS) 44°C for 6 h. (*n* = 2) (B) Phenotype of silencing of pTRV‐PDS showing functional VIGS system. (C, D) Expression of pTRV2 in ERF‐silenced plants (C) WT and (D) *hsfa2* background. (E) Expression level of all *SlERFs* in each ERF‐silenced plant. Error bars represent SD (*n* = 3). Statistical significance was calculated using a student's *t*‐test *P < 0.05.
**Figure S16.** Survival rate of ERF‐silenced plants (*n* = 2).
**Figure S17.** Luciferase reporter assay showing activation of *SlBAG6* promoter by SlHSFA2 and SlERF4 or SlERF1.1 in 37°C in *N. benthamiana* leaves. Error bars represent SD (*n* = 3). Statistical significance was calculated using a student's *t*‐test ***P < 0.001.
**Table S8.** Oligos used in this study.

**Table S1.** Significantly differentially expressed genes (pBH <0.05) identified from pairwise comparisons WT_10C versus WT_10PT, hsfa2_10C versus hsfa2_10PT, and WT_10PT versus hsfa2_10PT.
**Table S2.** Gene ontology (GO) term enrichment analysis of upregulated and downregulated genes performed with g:Profiler.
**Table S3.** Upregulated thermal memory genes showing differential expression in 5 C versus P and 10 C versus PT comparisons.
**Table S4.** Downregulated thermal memory genes showing differential expression in 5 C versus P and 10 C versus PT comparisons.
**Table S5.** Heat shock element (‐GAANNTTC‐) motifs in upregulated memory genes.
**Table S6.** Heat shock element (‐GAANNTTC‐) motifs in downregulated memory genes.
**Table S7.** Significantly differentially expressed genes (pBH <0.05) in WT P + T versus WT NHS and hsfa2 P + T versus hsfa2 NHS plants.

## Data Availability

RNA‐seq datasets generated in this study from shoot apices of WT plants after priming and triggering treatments, as well as datasets from WT and *hsfa2* mutant plants after triggering HS, are available in the NCBI Sequence Read Archive (SRA; https://www.ncbi.nlm.nih.gov/sra) under project IDs PRJNA1373253.
