# Supplementary material for: Thermal priming enhances yield after recurrent heat stress and involves SlHSFA2‐SlERFs in the regulation of transcriptional thermal memory in tomato
Source: Plant J. 2026 Oct 8;128(1):e71162. doi: 10.1111/tpj.71162 (PMC13646866; doi:10.1111/tpj.71162)
Supplement: Supplementary file 1 [file TPJ-128-0-s002.docx]

## **Supplementary Information**

**Thermal priming enhances yield after recurrent heat stress and involves SlHSFA2-SlERFs in regulation of transcriptional thermal memory in tomato**

Debnath P., Korkuc P., Kumar K., Bueno P.C.P., Jaeger K., Abel C., van Dam N.M., Wigge P.A., Olas J.J.

**Figure S1.** Thermopriming phenotype of tomato wild-type plants with different memory lengths grown under long day photoperiod (16 h light and 8 h darkness). **(A)** Thermopriming phenotype of non-stressed control (NHS), primed (P), primed+triggered (P+T), and triggered (T) tomato plants after a memory phase of 3 days**. (B)** Survival rate of WT plants in panel (A) and (C-E). **(C-E)** Thermopriming phenotype of non-stressed control (NHS), primed (P), primed+triggered (P+T), and triggered (T) tomato plants after a memory phase of (C) 10 days, (D) 20 days, or (E) 25 days. (**F, G)** Shoot fresh weight, and shoot length of NHS, P, P+T, and T tomato plants with 5-day memory phase. Error bars represent s.d. (*n*=5, except for T-only plants where *n*=4). Statistical significance was calculated using a student’s *t*-test **p*<0.05; ***p*<0.01; ****p*<0.001. (**H)** Trypan blue stationing of NHS, P, P+T, and T tomato seedlings with a five-day memory phase performed 24 h after triggering stress.

**Figure S2.** Detailed phenotypic characterization of control (NHS), primed-only (P), primed+triggered (P+T) tomato wild-type plants grown in long day (16 h light and 8 h darkness) under controlled conditions and exposed to thermopriming treatment with a five-day memory phase**. (A)** qRT-PCR analysis showing the expression of *CLAVATA1* (*SlCLV1*) in meristems of NHS and primed plants at 0.5 and 2 h after priming, and in meristems of NHS, P+T, and T-only plants at 0.5 and 6 h after triggering. Error bars represent s.d. (*n*=2). Statistical significance was determined using a student’s *t*-test **p*<0.05; ***p*<0.01; ns (not significant). (**B, C)** Yield phenotype of NHS and P+T plants from a second independent experiment, assessed 60 days after first flower appearance under controlled growth conditions. (**D)** Number of fruits produced by NHS and P+T plants at 60 days after the appearance of the first flower from a second independent experiment. (**E)** Total fruit weight of fruits produced by NHS and P+T plants grown in controlled growth chamber from a second independent experiment. Error bars in (D, E) represent s.d. (*n*=6 for NHS, and *n*=5 for P+T). (**F)** Ripening phenotype of NHS and P+T fruits. (G) Plant height of one-month-old NHS, P-only, and P+T tomato plants grown under controlled conditions. (n=5). (H) Phenotypes of one-moth-old NHS, P-only, and P+T plants grown under controlled conditions. Statistical significance was calculated using a student’s *t*-test **p*<0.05; ***p*<0.01; ****p*<0.001.

**Figure S3.** The average air temperature recorded during field experiment (from July till September) using the weather station collocated at the field.

**Figure S4.** Phenotype of control (NHS) and primed+triggered (P+T) tomato wild-type plants with a five-day memory phase grown under non-controlled conditions (field experiment). Priming was performed at five days after germination (DAG) and triggering treatment at 10 DAG. Plants were transferred to small pots at 13 DAG (3 days after triggering; DAT), moved to an open greenhouse for acclimatization at 20 DAG (10 DAT), and finally transplanted to the field at 27 DAG (17 DAT). Experiment was performed once.

**Figure S5**. Phenotypic characterization of non-stressed control (NHS) and primed+triggered wild-type tomato plants grown under non-stressed conditions (field experiment). (**A)** Plant height of NHS and P+T plants over time in the field experiment. (**B)** Root phenotype of NHS and P+T plants on the day of transfer to soil (field; 27 days after germination (DAG)/ 20 DAT (days after triggering). (**C, D)** Root fresh weight (C) and dry weight (D) of NHS and P+T plants analysed at 27 DAG/20 DAT. (**E)** Diameter of mature red fruits. (**F, G)** Number of seeds produced by NHS and P+T plants. Error bars represent s.d. Statistical significance was calculated using a student’s *t*-test **p*<0.05, ****p*<0.001.

**Figure S6.** RNA-seq analysis of control (NHS), primed (P), primed+triggered (P+T), and triggered (T) wild-type tomato plants with a five-days memory phase. **(A)** Principal component analysis (PCA) of TPM values showing clustering of meristem samples of NHS (grey), P (orange), P+T (red), and T-only (black) plants during the thermomemory. The 5NHS samples serve as controls for the 5P samples, whereas the 10NHS samples correspond to the P+T and T samples. (**B)** Heatmap of TPM values showing the average correlation among all samples. (**C, D)** Expression level of *HEAT SHOCK PROTEIN 17.6* (*SlHSP17.6*) and *HEAT SHOCK FACTOR A2* (*SlHSFA2*) analysed at the shoot apex of NHS and P-only plants at 0.5, 2, 24, 72, and 120 h after priming. Error bars represent s.d. Statistical significance was calculated using a student’s *t*-test ****p*<0.001.

**Figure S7. Transcriptional thermal memory genes**. **(A)** Venn diagram of significantly upregulated DEGs (p_BH<0.05) in P *versus* NHS at 0.5 h after priming and in P+T *versus* NHS at 0.5 h after triggering. **(B)** Venn diagram of significantly upregulated DEGs (p_BH<0.05; Log2FC≥1) in P *versus* NHS at 0.5 h after priming and in P+T *versus* NHS at 0.5 h after triggering. **(C)** Heatmap showing the expression of differentially expressed *HEAT SHOCK FACTORs* (*HSF*s) genes after priming and triggering, presented as Log2 fold change (log2 FC). **(D)** Expression level of thermal memory genes analyzed with *Clathrin Adaptor Complexes medium subunit (SlCAC)* as housekeeping gene. Error bars represent s.d. Statistical significance was calculated using a student’s *t*-test ***p*<0.01, ****p*<0.001.

**Figure S8.** Relative expression of *WUSCHEL* (*SlWUS*) analysed at the shoot apex of NHS and P+T WT and *hsfa2* mutant plants at 0.5 h after triggering. Error bars indicate s.d. (n=3). Statistical significance was calculated using Student *t*-test **p*<0.05; ***p*<0.01.

**Figure S9. RNA-seq analysis of non-heated (NHS) and primed+triggered (P+T) WT and *hsfa2* mutant plants at 0.5 h after triggering. (A)** Principal component analysis (PCA) of TPM values showing clustering of meristem samples of NHS (C, grey), and P+T (PT, red) plants at 0.5 h after triggering. The 10C samples serve as controls for the 10PT samples. (B, C) Numer of differentially expressed genes (DEGs) in the shoot apex of NHS and P+T WT and *hsfa2* mutant plants with log_2_FC≥|1| (B) or log_2_FC≥|2| (C).

**Figure S10.** Heatmap of genes common between transcriptional upregulated thermal memory genes (267 genes) and differentially expressed genes (2825 genes; Log2FC≥1, pBH<0.05) in *hsfa2* NHS vs. *hsfa2* P+T 0.5 h after triggering, based on VST values. Note that 9 genes did not show a transcriptional memory pattern in second independent RNA-seq experiment and were therefore removed from the heatmap resulting in 83 genes. First, mean values per group were calculated, and then z-scores were computed per gene.

**Figure S11.** Expression level of memory genes. Expression level of *B-CELL LYMPHOMA 2* (*Bcl-2*)-*ASSOCIATED ATHANOGENE* *5* (*SlBAG5*), *HEAT SHOCK PROTEIN 17.6* (*SlHSP17.6*) and *ETHYLENE RESPONSIVE FACTOR 1.1.* (*SlERF1.1*) at the shoot apex of control, not-stressed (NHS) and primed+triggered (P+T) wild-type (WT) and *hsfa2* mutant plants. Error bars represent s.d. (*n*=3). Statistical significance was calculated using a student’s *t*-test **p*<0.05.

**Figure S12.** Luciferase reporter assay and quantifications showing activation of *SlERF1.1* promoter by SlHSFA2 in 37°C in *N. benthamiana* leaves. The quantification is presented as LUC/REN. Error bars represent s.d. (*n*=6). Statistical significance was calculated using a student’s *t*-test ns, not significant.

**Figure S13.** Heatmap showing the expression of ethylene-related genes with log_2_ fold change (log_2_FC) > 3 in primed+triggered (P+T) wild-type (WT) tomato and *hsfa2* mutant plants after triggering stress. Expression values were calculated from RNA-seq data and are shown as log_2_FC relative to non-stress controls (NHS). Red colour indicted upregulation and blue downregulation.

**Figure S14.** SlERFs and SlHSFA2 colocalization and interaction. **(A)** Colocalization of *35S::HSFA2-mTurquoise* and various *35S::ERFs-mVenus* in *N. benthamiana* leaves at 37°C. (**B)** Yeast two-hybrid (Y2H) assay showing SlERFs fused to the GAL4-activation domain co-transformed with the empty GAL4 binding domain (BD) vector. Yeast cells were grown on selective media (SD/-Leu/-Trp) and (SD/-Leu/-Trp/-His/-Ade). (**C)** Bimolecular fluorescence complementation (BiFC) assay showing interaction between SlHSFA2 and SlERF.C6., SlERF1.2, SlAP2-like factor, and SlMBF1-ER24 at 37 °C in *N. benthamiana* leaves. Note that constructs expressing SlHSFA2 fused to the N-terminal half of YFP and other protein fused to C-terminal half of YFP.

**Figure S15.** SlERFs-silenced plants. (**A)** Relative transcript level of *ETHYLENE RESPONSIVE FACTORS* (*SlERFs*) measured in ERFs-silenced and TRV-EV control plants exposed to heat stress (HS) 44 ºC for 6 h. (*n*=2) **(B)** Phenotype of silencing of pTRV-PDS showing functional VIGS system. **(C, D)** Expression of pTRV2 in ERF-silenced plants (C) WT and (D) *hsfa2* background. **(E)** Expression level of all *SlERFs* in each ERF-silenced plant. Error bars represent s.d.. (*n*=3) Statistical significance was calculated using a student’s *t*-test **p*<0.05.

**Figure S16.** Survival rate of ERF-silenced plants. (n=2).

**Figure S17.** Luciferase reporter assay showing activation of *SlBAG6* promoter by SlHSFA2 and SlERF4 or SlERF1.1 in 37°C in *N. benthamiana* leaves. Error bars represent s.d. (*n*=3). Statistical significance was calculated using a student’s *t*-test ****p*<0.001.

**Table S8**. Oligos used in this study.

| **Gene** | **Oligos name** | **Sequence** |
| --- | --- | --- |
| **RNA in situ Hybridization** | | |
| *SlHSFA2* | HSFA2 F in-situ  HSFA2 R in-situ | ATGGAGGATGTAGTGAAAGTG  TTAACCCCATTCAGGTGTTTTC |
| *SlCLV3* | CLV3 F in-situ  CLV3 R in-situ | ATGTCTTTGATCAATGCTAAATATTTC  TTATGGAGTCTTAGGTTTCTTAGG |
| *SlSTM* | STM F in-situ  STM R in-situ | ATGGAGGGTGGTTCTAGTGG  TCAGAGGAGAGAGGGTGTCATATC |
| *SlHSP17.6* | HSP17.6 F in-situ  HSP17.6 R in-situ | ATGGCAGTTGTTCAAATGGCTG  TTAGTCCACTTCTTCCATCTTGC |
|  | SP6 promoter F  T7 promoter R | ATTTAGGTGACACTATAGA  TAATACGACTCACTATA |
| **qRT-PCR** | | |
| *SlAP2-like factor* | AP2_qF  AP2_qR | GGCAGGGTGTTTGGAAATAAG  GTTTGGTCCACGGTACTGTAT |
| *SlERF1.2* | ERF1.2_q  ERF1.2_qR | TTCCTGAACTAGCCGGTTTAC  CATCATCGAACTTGTCCATTGC |
| *SlHSP17.6* | HSP17.6 qF  HSP17.6 qR | CTCGTAGCGGAGAAGGTTATTG  CTCCACTGACATCCCTTGTAAC |
| *SlCLV3* | CLAVATA3 qF  CLAVATA3 qR | GCTGCATTTGGGAAGGTATACTA  ATGCAATGGATCAGGACCAG |
| *SlCLV1* | CLAVATA1 qF  CLAVATA1 qR | TGTGTACCGAGGGTCTATGT  CCGGATCCTTCCTAGTGTTTG |
| *SlWUS* | WUSCHEL qF  WUSCHEL qR | GGCTATGGAGAAGAGCTTTAGG  GGGTAAGTTGCTGGAGAAGTAG |
| *SlSTM* | STM qF  STM qR | TCCTCACTACCATCGTCTCTT  TGCTGACGTGGCACATATT |
| *SlBAG6* | BAG6 qF  BAG6 qR | TGTGCTTACCACCTGATGAC  GGATTCTCCTCCTGCTCTTTAC |
| *SlERF4* | ERF4 qF  ERF4 qR | TCGTCGTTAGGAACGGTTAATG  GTGACGATGGTGGTGGTATAAA |
| *SlACTIN* | Actin qF  Actin qR | AGGAACACCCTGTTCTCCTGACTG  CAGAGAAAGCACAGCCTGGATAG |
| *SlCAC* | CAC qF  CAC qR | CCTCCGTTGTGATGTAACTGG  ATTGGTGGAAAGTAACATCATCG |
| **BiFC** | | |
| *SlAP2-like factor* | AP2_BifC_F  AP2_BifC_R | gtataataaagttgctagcgatggcgaaaacctcaaaattg  agctgggtagccggcctgcaatcagaggtctcgagttc |
| *SlERF1.2* | ERF1.2_BifC_F  ERF1.2_BifC_R | gtataataaagttgctagcgATGAATTCAGTAGCAGCG  agctgggtagccggcctgcaATAATTCCACAACAAAGAAGG |
| *SlBAG6* | BAG6_BifC_F  BAG6_BifC_R | gtataataaagttgctagcgATGTATCCTACGAATCCTTACC  agctgggtagccggcctgcaCATTGCCAACCCCACATTC |
| *SlMBF1-ER24* | MBF1_BifC_F  MBF1_BifC_R | gtataataaagttgctagcgATGCCGATGCGACCAACAG  agctgggtagccggcctgcaTGACTTGTGAATTTTACCTCTAAGTTTAACAC |
| *SlERF1.1* | ERF1.1_BifC_F  ERF1.1_BifC_R | gtataataaagttgctagcgATGATGTTACCTATGGATTATAC  agctgggtagccggcctgcaGAAATCCAAGACATCCAAC |
| *SlERF1a* | ERF1a_BifC_F  ERF1a_BifC_R | gtataataaagttgctagcgATGGGCTCTTCACAAGAG  agctgggtagccggcctgcaAAGTAAATCAAAAATGCCTTTTC |
| *SlERF4* | ERF4_BifC_F  ERF4_BifC_R | gtataataaagttgctagcgATGGTGAAACCCAATTCG  agctgggtagccggcctgcaAAAATTCCAAAGAAATGAAGTC |
| *SlERF1b* | ERF1b_BifC_F  ERF1b_BifC_R | gtataataaagttgctagcgATGAGTATTGTAATTGATGATGATG  agctgggtagccggcctgcaCATTTGTATTAACATTTGTAAATCC |
| *SlHSFA2* | HSFA2_BifC_F  HSFA2_BifC_R | tttgtacaaaaaagcaggctATGGAGGATGTAGTGAAAG  agttgggtggccggcctgcaACCCCATTCAGGTGTTTTC |
| *SlERF.C.6* | ERF.C.6_BifC_F  ERF.C.6_BifC_R | gtataataaagttgctagcgATGGGTTCTCCACAAGAG  agctgggtagccggcctgcaTATCATAACAAGCTGAGATAATG |
| **VIGS** | | |
| *SlERF1.1* | ERF1.1 VIGS F  ERF1.1 VIGS R | ttctgtgagtaaggttaccggaatctcccggactattac  gacgcgtgagctcggtaccgctcagaggtgtaaaagtc |
| *SlBAG6* | BAG6 VIGS F  BAG6 VIGS R | ttctgtgagtaaggttaccgatgtatcctacgaatccttac  gacgcgtgagctcggtaccgagtaggatacatcacagg |
| *SlERF4* | ERF4 VIGS F  ERF4 VIGS R | ttctgtgagtaaggttaccgatcggaaattagattacctaatagtag  gacgcgtgagctcggtaccgggtgattccgctcgaaatatttc |
| *SlERF1b* | ERF1b VIGS F  ERF1b VIGS R | ttctgtgagtaaggttaccgtgatgatgatgaaatcttctc  gacgcgtgagctcggtaccgcctctcctatttggatctc |
| *SlERF1a* | ERF1a VIGS F  ERF1a VIGS R | ttctgtgagtaaggttaccgatgggctcttcacaagag  gacgcgtgagctcggtaccgcttcttctcactctcaacg |
| *SlPDS* | PDS VIGS F  PDS VIGS R | ttctgtgagtaaggttaccgatgcctcaaattggacttg  gacgcgtgagctcggtaccgaccagtctcgtaccaatc |
| **LUC reporter assay** | | |
| *SlERF1.1* | pERF1.1 LUC F  pERF1.1 LUC R | cgaattcctgcagcccggggGGATGATGGAATTGGCATTGCG  cggccgctctagaactagtgGAAGTTCGATCAAGCACAGC |
| *SlBAG6* | pBAG6 LUC F  pBAG6 LUC R | cgaattcctgcagcccggggCTCACTCTACACCACATATTG  cggccgctctagaactagtgCCAAATAACTCAATTTCCAGCAC |
| *SlHSFA2* | HSFA2 3933 F  HSFA2 3933 R | ggagcgggagcaggcgggatggaggatgtagtgaaag  gcggggtaccagatctgttaaccccattcaggtgttttc |
| *SlERF1.1* | ERF1.1_3933 F  ERF1.1_3933 R | ggagcgggagcaggcggATGATGTTACCTATGGATTATACAAGG  GCGGGGTACCAGATCTGTTAGAAATCCAAGACATCCAACTC |
| *SlERF1a* | ERF1a_3933 F  ERF1a_3933 R | ggagcgggagcaggcggATGGGCTCTTCACAAGAG  GCGGGGTACCAGATCTGTCAAAGTAAATCAAAAATGCC |
| *SlERF4* | ERF4_3933 F  ERF4_3933 R | ggagcgggagcaggcggATGGTGAAACCCAATTCGAAAG  GCGGGGTACCAGATCTGTCAAAAATTCCAAAGAAATGAAGTCCCC |
| *SlERF1b* | ERF1b_3933 F  ERF1b_3933 R | ggagcgggagcaggcggATGAGTATTGTAATTGATGATGATG  GCGGGGTACCAGATCTGTCACATTTGTATTAACATTTGTAAATCC |
| **Y2H** | | |
| *SlERF1b* | ERF1b AD F  ERF1b AD R | TATGGCCATGGAGGCCAGTGAATTCATGAGTATTGTAATTGATGATGATG  TCTGCAGCTCGAGCTCGATGGATCCCTCACATTTGTATTAACATTTGTAAATCC |
| *SlERF1b* | ERF1b BD F  ERF1b BD R | CATATGGCCATGGAGGCCGAATTCATGAGTATTGTAATTGATGATGATG  gcggccgctGCAGGTCGACGGATCCTCACATTTGTATTAACATTTGTAAATCC |
| *SlERF4* | ERF4 AD F  ERF4 AD R | TATGGCCATGGAGGCCAGTGAATTCATGGTGAAACCCAATTCGAAAG  TCTGCAGCTCGAGCTCGATGGATCCCTCAAAAATTCCAAAGAAATGAAGTCCCC |
| *SlERF4* | ERF4 BD F  ERF4 BD R | CATATGGCCATGGAGGCCGAATTCATGGTGAAACCCAATTCGAAAG  gcggccgctGCAGGTCGACGGATCCTCAAAAATTCCAAAGAAATGAAGTCCCC |
| *SlERF1.1* | ERF1.1 AD F  ERF1.1 AD R | TATGGCCATGGAGGCCAGTGAATTCATGATGTTACCTATGGATTATACAAGG  TCTGCAGCTCGAGCTCGATGGATCCCTTAGAAATCCAAGACATCCAACTC |
| *SlERF1.1* | ERF1.1 BD F  ERF1.1 BD R | CATATGGCCATGGAGGCCGAATTCATGATGTTACCTATGGATTATACAAGG  gcggccgctGCAGGTCGACGGATCCTTAGAAATCCAAGACATCCAACTC |
| *SlERF1a* | ERF1a AD F  ERF1a AD R | TATGGCCATGGAGGCCAGTGAATTCATGGGCTCTTCACAAGAG  TCTGCAGCTCGAGCTCGATGGATCCCTCAAAGTAAATCAAAAATGCC |
| *SlERF1a* | ERF1a BD FP  ERF1a BD RP | CATATGGCCATGGAGGCCGAATTCATGGGCTCTTCACAAGAG  gcggccgctGCAGGTCGACGGATCCTCAAAGTAAATCAAAAATGCC |
| *SlHSFA2* | HSFA2 BD F  HSFA2 BD R | CATATGGCCATGGAGGCCGAATTCATGGAGGATGTAGTGAAAGTGAAG  gcggccgctGCAGGTCGACGGATCCTTAACCCCATTCAGGTGTTTTCAC |
| *SlHSFA2* | HSFA2 AD F  HSFA2 AD R | TATGGCCATGGAGGCCAGTGAATTCATGGAGGATGTAGTGAAAGTGAAG  TCTGCAGCTCGAGCTCGATGGATCCCTTAACCCCATTCAGGTGTTTTCAC |
| *SlERF.C.6* | ERF.C.6 AD F  ERF.C.6 AD R | TATGGCCATGGAGGCCAGTGAATTCATGGGTTCTCCACAAGAGACTTG  TCTGCAGCTCGAGCTCGATGGATCCCTTATATCATAACAAGCTGAGATAATGG |
| *SlERF.C.6* | ERF.C.6 BD F  ERF.C.6 BD R | CATATGGCCATGGAGGCCGAATTCATGGGTTCTCCACAAGAGACTTG  gcggccgctGCAGGTCGACGGATCCTTATATCATAACAAGCTGAGATAATGG |
| **Colocalization** | | |
| *SlHSFA2* | HSFA2_F  HSFA2_R | GGACGAGCTCGGTACCCGGGATGGAGGATGTAGTGAAAG  cctcgcccttgctcaccatGCCGCCGCCACCCCATTCAGGTGTTTTC |
| *SlMBF1-ER24* | MBF1_F  MBF1_R | GGACGAGCTCGGTACCCGGGATGCCGATGCGACCAACAG  cctcgcccttgctcaccatGGAACCTGATCCTGACTTGTGAATTTTACCTCTAAGTTTAACAC |
| *SlERF.C.6* | ERF.C.6_F  ERF.C.6_R | GGACGAGCTCGGTACCCGGGATGGGTTCTCCACAAGAG  cctcgcccttgctcaccatGGAACCTGATCCTATCATAACAAGCTGAGATAATG |
| *SlERF1b* | ERF 1b_F  ERF 1b_R | GGACGAGCTCGGTACCCGGGATGAGTATTGTAATTGATGATGATG  cctcgcccttgctcaccatGGAACCTGATCCCATTTGTATTAACATTTGTAAATCC |
| *SlERF1a* | ERF1a_F  ERF1a_R | GGACGAGCTCGGTACCCGGGATGGGCTCTTCACAAGAG  cctcgcccttgctcaccatGGAACCTGATCCAAGTAAATCAAAAATGCCTTTTCCATCTCC |
| *SlERF1.1* | ERF1.1_F  ERF1.1_R | GGACGAGCTCGGTACCCGGGATGATGTTACCTATGGATTATAC  cctcgcccttgctcaccatGGAACCTGATCCGAAATCCAAGACATCCAAC |
| *SlERF4* | ERF4_F  ERF4_R | GGACGAGCTCGGTACCCGGGATGGTGAAACCCAATTCG  cctcgcccttgctcaccatGGAACCTGATCCAAAATTCCAAAGAAATGAAGTCCCCTG |
